# Selected I-III-VI_2_ Semiconductors: Synthesis, Properties and Applications in Photovoltaic Cells

**DOI:** 10.3390/nano13212889

**Published:** 2023-10-31

**Authors:** Shubham Shishodia, Bilel Chouchene, Thomas Gries, Raphaël Schneider

**Affiliations:** 1 Université de Lorraine, CNRS, LRGP, F-54000 Nancy, France; shubham.shishodia@univ-lorraine.fr (S.S.); bilel.chouchene@univ-lorraine.fr (B.C.); 2 Université de Lorraine, CNRS, IJL, F-54000 Nancy, France; thomas.gries@univ-lorraine.fr

**Keywords:** quantum dots, I-III-VI_2_ group, synthesis, photosensitizers, photovoltaic cell

## Abstract

I–III–VI_2_ group quantum dots (QDs) have attracted high attention in photoelectronic conversion applications, especially for QD-sensitized solar cells (QDSSCs). This group of QDs has become the mainstream light-harvesting material in QDSSCs due to the ability to tune their electronic properties through size, shape, and composition and the ability to assemble the nanocrystals on the surface of TiO_2_. Moreover, these nanocrystals can be produced relatively easily via cost-effective solution-based synthetic methods and are composed of low-toxicity elements, which favors their integration into the market. This review describes the methods developed to prepare I-III-VI_2_ QDs (AgInS_2_ and CuInS_2_ were excluded) and control their optoelectronic properties to favor their integration into QDSSCs. Strategies developed to broaden the optoelectronic response and decrease the surface-defect states of QDs in order to promote the fast electron injection from QDs into TiO_2_ and achieve highly efficient QDSSCs will be described. Results show that heterostructures obtained after the sensitization of TiO_2_ with I-III-VI_2_ QDs could outperform those of other QDSSCs. The highest power-conversion efficiency (15.2%) was obtained for quinary Cu-In-Zn-Se-S QDs, along with a short-circuit density (*J_SC_*) of 26.30 mA·cm^−2^, an open-circuit voltage (*V_OC_*) of 802 mV and a fill factor (*FF*) of 71%.

## 1. Introduction

Quantum dots (QDs) are zero-dimensional semiconductor nanocrystals that have gained high attention for decades. QDs exhibit unique properties including wide UV-visible absorption, tunable bandgap energy due to the quantum confinement effect, sharp photoluminescence (PL) emission and high-PL quantum yield (QY) [1,2,3]. These unique properties made QDs of high potential for various optical and optoelectronic applications like light-emitting diodes (LEDs) [4,5], photovoltaic (PV) cells [6,7], bio-imaging and sensing [8,9]. During the past decades, most of the studies were devoted to binary QDs such as CdSe, CdTe, PbS or PbSe due to their size and shape dependent optoelectronic properties and their relatively easy synthesis. However, these QDs contain toxic elements (Cd, Pb) which restrict the use of these nanocrystals for numerous biological and commercial applications [10]. Thus, the synthesis of low-toxic QDs has attracted high attention in the last few years.

I-III-VI_2_ semiconductor nanocrystals are composed of benign elements and constitute greener alternatives to II-VI (CdSe, CdTe,…) and IV-VI (PbS, PbSe,…) QDs [11,12,13,14,15,16]. Due to their large absorption coefficient, direct bandgap, strong PL emission in the visible and the near-infrared (NIR) light region and high-PL lifetimes, AgInX_2_ and CuInX_2_ (X = S or Se) have been extensively studied for various applications like PV cells [17,18]. The energy bandgap of these nanocrystals and thus their UV-visible absorption and their PL emission can easily be tuned by varying the size of the nanocrystals as well as their composition. Indeed, I-III-VI_2_ QDs can be prepared in both stoichiometric (AgInS_2_, AgInSe_2_, …) and non-stoichiometric compositions, and this compositional tunability is a key parameter for adapting their properties to the desired application and promoting their integration into numerous devices [11,12,13,14,15,16,17,18]. Moreover, I-III-VI_2_ QDs can form solid solutions with II-VI group semiconductors like ZnS, which further allows us to tune the energy bandgap [11,12,13,14,15,16,17,18].

Recent reviews summarize the synthesis and properties of AgInX_2_ and CuInX_2_ (X = S or Se) QDs and their applications, and these nanocrystals will not be the subject of this review [19,20,21,22,23,24]. The structural adaptability of I-III-VI_2_ semiconductors has led to the development of a large panel of nanocrystals, among which we can mention Ag(Cu)GaX_2_, Ag(Cu)InGaX_2_ or CuAlS_2_ QDs, whose properties are often complementary to those of AgInX_2_ and CuInX_2_ QDs. In this review, we describe the synthesis of Ag(Cu)GaX_2_, Ag(Cu)InGaX_2_ and CuAlS_2_ QDs and of the solid solution obtained after alloying with ZnS or Ga_2_S_3_; the properties of these nanocrystals; and their use as photoabsorbers in PV cells.

## 2. I-III-VI_2_ QD Optical Properties

**Figure 1:**
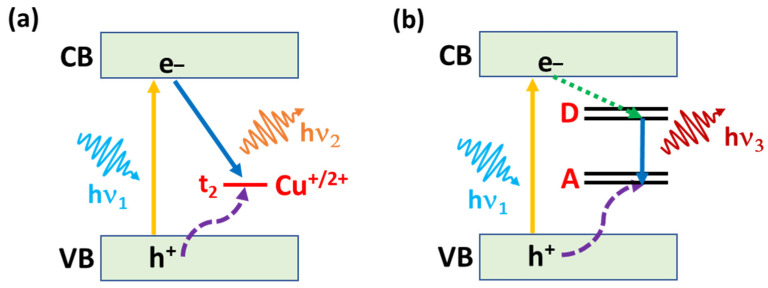
Illustration of the PL emission mechanisms in CuInS_2_ QDs (**a**) the “self-trapped” model and (**b**) the DAP mechanism. Solid lines are associated with the absorption or emission of a photon while dashed lines are associated with non-radiative processes.

I-III-VI_2_ QDs exhibit tail absorption, long PL emission lifetimes (a few hundreds of ns) and a broad PL emission, which depend on the metal ratio (for example, the Ag/In molar ratio) but not on the nanocrystal shape. A large Stokes shift is observed, indicating that the PL emission is not a band-edge emission between the valence band (VB) maximum and the conduction band (CB) minimum. The broadness of the UV-visible absorption and of the PL emission spectra originates from the trapping of electrons and holes in randomly located local states within the bandgap and not from the polydispersity in size and composition of the dots [15,16,17,18,19,20,21,22,23,24,25,26,27,28,29,30]. The “self-trapped” model has been proposed for PL emission [31]. Taking CuInS_2_ QDs as an example, holes generated in the VB after light excitation are captured by intragap Cu-related states, while electrons are mainly located at the CB edge. The charge-transfer recombination occurs between an electron in the CB with a Cu-localized hole (Figure 1a). Because I-III-VI_2_ QDs are usually prepared using off-stoichiometry, the lattice is rich in crystallographic defects (interstitial atoms, vacancies and anti-site defects). The PL emission of these QDs is therefore dominated by radiative recombination linked to donor-acceptor defects but also by surface-related defects due to the high surface-to-volume ratio of these nanocrystals (Figure 1b). Noteworthy is that the PL emission originating from intrinsic defects is significantly higher in intensity than that associated with surface defects, which mainly gives rise to non-radiative recombination pathways. In a donor-acceptor pair (DAP) recombination, a photoexcited electron trapped by a donor recombines with a hole trapped by an acceptor. This conducts to the emission of a photon with an energy depending on the distance r between the donor and the acceptor due to the Coulomb interaction between the ionized donor and acceptor formed after the charge recombination. The energy of the photon emitted is given by Equation (1):

(1) $${h\nu }_{\left(D^{0}-A^{0}\right)}=E_{g}-\left(E_{D}+E_{A}\right)+\frac{e^{2}}{4\;\pi \epsilon \epsilon_{0}R}-m\hbar \omega_{LO}$$

where *D*^0^ and *A*^0^ are the donor and the acceptor in the ground state, *E_g_* is the bandgap energy, *E_a_* and *E_d_* the binding energies of the acceptor and of the donor, respectively, *e* is the electron charge, ϵ_0_ the vacuum permittivity and ϵ the low-frequency relative dielectric constant of the semiconductor. *R* is the distance between the acceptor and the donor in the Coulombic interaction term and is determined using the crystal structure and the lattice constant. The last term corresponds to longitudinal optical (LO) phonon replicas. Consequently, higher-energy photons emitted by the QDs arise from donors in close proximity to acceptors, while lower-energy photons can be associated with DAPs spatially further apart.

## 3. I-III-VI_2_ QDs Synthesis

**Figure 2:**
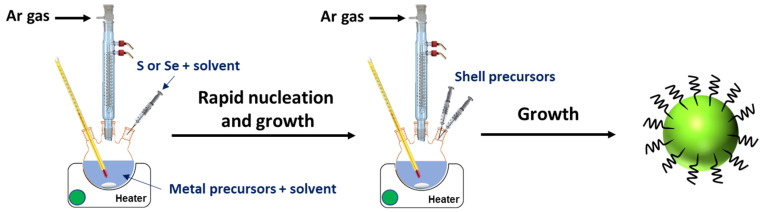
Schematic representation of I-III-VI_2_ QDs synthesis via the “hot-injection” method.

Due to their high formation energy, I-III-VI_2_ QDs are usually prepared at high temperatures in high-boiling-point solvents like oleylamine (OAm) or oleic acid (OA), which also acts as a capping ligand, or in a non-coordinating solvent like 1-octadecene (ODE). Dodecanethiol (DDT) is used as a ligand, and an S source is also commonly used. Aqueous-phase methods like hydrothermal synthesis or microwave-assisted synthesis have also been reported. The synthesis processes include:

- The “hot-injection method”, which is the most commonly used [20,32]. The synthesis usually begins with the injection of the S (or Se or Te) precursor into a hot solution of the metal precursors, which causes the fast formation of nuclei and therefore good control of the growth of the nanocrystals (Figure 2). The growth of QDs via Ostwald ripening can be conducted at a lower temperature, so as not to generate new nuclei. This process allows for the production of high-quality and nearly monodisperse nanocrystals and is well-suited for the preparation of core/shell QDs.

- The “non-injection” method involves the mixing of precursors followed by heating at a specific temperature at which the decomposition of precursors takes place and produces nanocrystals [33,34]. This method allows for a relatively good control of the nucleation and of the growth of the dots and thus of their average size and composition.
- Single-source thermal decomposition usually involves the mixing of precursors (for example, copper and indium diethyldithiocarbamate or the (PPh3)2CuIn(SEt)4 complex) in OAm followed by their heating at a high temperature to break down the precursors into Cu^+^, In^3+^ and S^2−^, thus allowing for the nucleation of CuInS_2_ QDs followed by their growth [35,36]. Single-source thermal decomposition is the easiest method to produce QDs, but it does not allow for precise control over QD composition and therefore usually does not lead to high-quality nanocrystals.
- Aqueous synthesis is less developed for the synthesis of I-III-VI_2_ QDs. It usually involves the injection of an S^2−^ or Se^2−^ precursor into an aqueous solution containing the metal precursors and the hydrophilic ligand (3-mercaptopropionic acid, glutathione, cysteine, …), followed by heating (reflux, hydrothermal, microwave, …) [14]. This method has many advantages, including a low cost and the use of water as a solvent, and allows for the production of QDs dispersible in water, which avoids a ligand exchange or an encapsulation in amphiphilic polymers for biological applications. However, the quality of QDs prepared in the aqueous phase is generally much lower than those produced in organic solvent, which limits their use in many applications. Note, however, that some of the best QDs prepared in the aqueous phase were obtained using microwave-assisted synthesis due to the volumetric heating that allows reactions to proceed faster compared to conventional hydrothermal synthesis [37,38].

**Figure 3:**
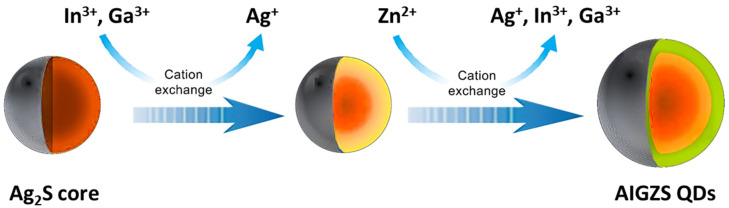
Schematic representation of the cation exchange during the synthesis of AIGZS QDs.

A phenomenon inherent to the synthesis of I-III-VI_2_ QDs is the cation exchange, in which metal cations present in the nanocrystal host lattice are replaced by cations in the solution [11,13,14]. For example, considering the preparation of quinary Ag-In-Ga-Zn-S (AIGZS) QDs, Ag^+^ ions react quickly with the S^2−^ precursor to form Ag_2_S. The incorporation of In^3+^ and Ga^3+^ into the nanocrystals requires higher temperatures (ca. 160 and 240 °C, respectively) (Figure 3). During the final ZnS shelling, Zn^2+^ ions diffuse into the Ag-In-Ga-S QDs and expel some Ag^+^, In^3+^ and Ga^3+^ cations to form gradient alloyed AIGZS QDs. These gradual exchanges taking place at well-defined temperatures avoid defects in the inhomogeneous nucleation process.

Multiple synthetic routes have been studied to synthesize QDs in aqueous or organic phases. High-temperature decomposition, or hot-injection, in an organic medium is the most commonly used process.

## 4. I-III-VI_2_ QDs and Derivatives: Synthesis and PV Applications

**Figure 4:**
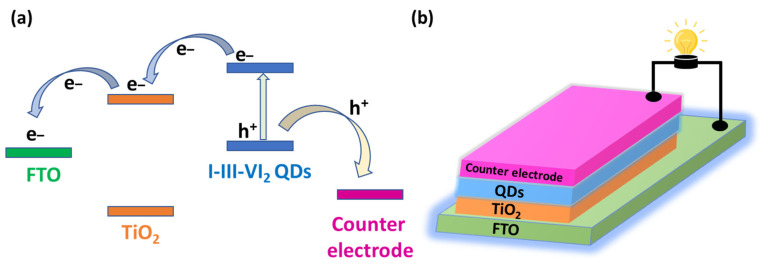
(**a**) Energy-level alignment and (**b**) charge-transfer process in QDSSCs.

Because PV cells allow us to convert solar energy into electricity, these devices represent some of the most promising technology to meet the ever-increasing energy demand. The light-harvesting efficiency of I-III-VI_2_ QDs is higher than that of TiO_2_ due to their small bandgap and large absorption coefficient, and the use of these nanocrystals is beneficial for PV cell engineering. Upon light irradiation, electron-hole pairs are generated in I-III-VI_2_ QDs. When in contact with TiO_2_, photoexcited electrons from QDs can transfer to the CB of TiO_2_, and then electrons are further transferred to the external circuit (for example, fluorine-doped tin oxide, FTO). Simultaneously, holes in the VB of QDs are transported to the counter electrode (for example, Au or Pt) to complete the circuit, resulting in the flow of electricity (Figure 4).

Despite of their disordered nature, thin films composed of I-III-VI_2_ materials have demonstrated high potential for thin-film-based PV cells with efficiencies up to 22% due to their high absorption coefficients, relatively low cost and high stability [39]. For QD-sensitized solar cells (QDSSCs), good performance is usually dependent on the efficient electron injection from photoexcited QDs into the conduction band of TiO_2_ films. It is thus important to decrease both the internal charge recombination within the QDs (via the decrease in the defect density) and the interface recombination at the TiO_2_/QDs/electrolyte interface. For PV cell applications, QDs must meet the following criteria to avoid a sharp deterioration in performance:

- QDs should exhibit a narrow bandgap, allowing for the harvesting of light in the visible and NIR regions.
- The CB energy of QDs should be high to efficiently extract and transfer photogenerated electrons from QDs to TiO_2_. A large difference in energy between the CB of QDs and that of TiO_2_ promotes a fast extraction rate of photo-generated electrons.
- The density of defect trap states, especially deep-level trap states, should be low, as these defects will not only cause a quenching of photoexcited electrons before their transfer to TiO_2_ but also a back transfer of these electrons from TiO_2_, which causes the charge recombination loss.

The shelling of QDs with a wide-bandgap semiconductor like ZnS to remove surface-defect trap states could be a solution, but the ZnS shell may hinder the extraction of electrons [40]. The alloy formation seems to be more promising as it not only allows us to tune QDs’ CB energy, for example, by varying the Cu/In/Zn or Cu/Ga/Zn molar ratios used for the synthesis, but it also mitigates the density of surface-defect trap states. These various aspects will be discussed later in this article.

After depositing QDs on the TiO_2_ surface, the power-conversion efficiency (PCE) of the cells is determined using Equation (2):

(2) $$PCE=\frac{J_{sc}V_{oc}FF}{I_{0}}$$

where *J_sc_* is the short-circuit current density, *V_oc_* is the open-circuit voltage, *FF* is the fill factor, and *I*_0_ is the incident light intensity equal to 1 sun (1000 W m^−^^2^).

The external quantum efficiency (EQE) of a PV cell is the flux of electrons extracted from the solar cell under operating conditions divided by the flux of photons incident on the solar cell and can be determined using Equation (3):

(3) $$EQE\;\left(\lambda ,r,S_{gb}\right)=\frac{hcJ_{sc}}{e\lambda I_{0}(\lambda )}\;$$

where *h* is Planck’s constant, *c* is the velocity of light, *J_sc_* is the short-circuit current density, *e* is the charge of the electron, *λ* is the wavelength of the exciter light and *I*_0_(*λ*) is the electric current due to the photoelectric effect.

### 4.1. Cu-In-Se-Te (CISeTe) QDs

CuInTe_2_ is a semiconductor with a direct bandgap of 1.02 eV in the bulk state and thus exhibits high potential for PV applications. However, CITe QDs show modest stability due to the facile oxidation of Te atoms. Gradient alloyed CuInTe_2−x_Se_x_ QDs (Te-rich core) were developed due to their higher stability to effectively exploit the optical properties of these nanocrystals [41]. CISeTe QDs were prepared by the hot-injection method and showed a PL emission peak located at ca. 855 nm (*E_g_* = 1.45 eV). The trap emission could be suppressed by locating Se atoms near the surface of the dots, while Te atoms were located at the core and thus protected from oxidation. Alloyed CISeTe QDs were coated on TiO_2_ and used as photosensitizers in PV cells. The cell was demonstrated to absorb light up to 1000 nm and exhibits a *J_sc_* of 17.4 mA·cm^−2^, a *V_OC_* of 0.40 V, a *FF* of 44.1% and an overall PCE of 3.1%. More recently, the Te/Se ratio was optimized and the PCE could be increased to 3.75% using QDs with the CuInTe_1.__2_Se_0.__8_ composition (*J_sc_* = 11.707 mA·cm^−2^, *V_OC_* = 0.683 V, *FF* = 51.6%) [42].

### 4.2. Ag-In-Zn-Te (AIZTe) QDs

AIZTe nanocrystals can be prepared via the thermal decomposition method using AgOAc, In(OAc)_3_ and Zn(OAc)_2_ and Te as precursors [43]. Transmission electron microscopy (TEM) showed rod-shaped AIZTe particles with an average length of 17 nm and an average width of 4.5 nm. The optical properties of AIZTe nanocrystals could be tuned in the NIR region by varying the Ag/In/Zn/Te molar ratio. The results show that the absorption edge was shifted to the lower wavelengths (from 965 to 710 nm) with the increase in the Zn content, which originates from the increase in the *Eg* value from 1.20 to 1.60 eV. PL emission spectra show that the increase in Zn content was accompanied by a blue shift from 1010 to 809 nm, and the PL QY was decreased from 47 to 0.07% due to the decrease in radiative recombination rate.

**Figure 5:**
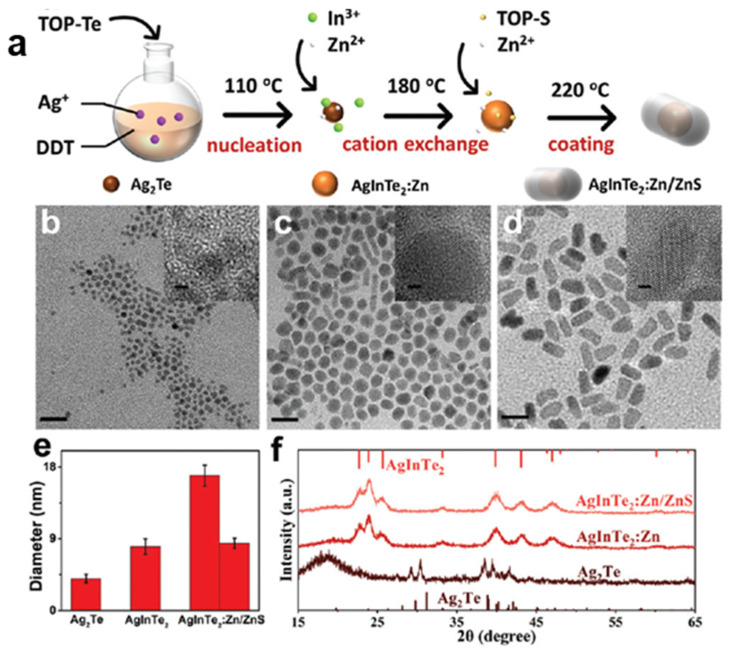
(**a**) Scheme for the synthesis of AgInTe_2_:Zn/ZnS NCs. TEM images of the as-synthesized (**b**) Ag_2_Te, (**c**) AgInTe_2_:Zn, and (**d**) AgInTe_2_:Zn/ZnS NCs (Scale bar = 20 nm). Insets show their corresponding high-resolution TEM images (Scale bar = 2 nm). (**e**) Size distribution histograms of the as-synthesized NCs through calculating 200 nanoparticles in their TEM images. (**f**) Powder XRD patterns of the as-synthesized Ag_2_Te, AgInTe_2_:Zn and AgInTe_2_:Zn/ZnS NCs, respectively. Used with permission from ref. [44]. Copyright 2022 RSC publishing.

The hot-injection method was recently used by Li et al. to prepare core/shell AIZTe/ZnS QDs [44]. The structural and microstructural characterizations confirm the preparation of AgTe, AIZTe and AIZTe/ZnS nanocrystals with an average size of 4.0, 8.0 and 16.9 nm, respectively. The X-ray diffractograms (XRD) showed a pure hexagonal phase (Figure 5). After shelling with ZnS, the PL lifetime decay was significantly increased from 116.8 to 373.4 ns for AIZTe and AIZTe/ZnS QDs, respectively. In addition, AIZTe/ZnS QDs showed good stability, which should favor the use of these nanocrystals in biomedical applications.

### 4.3. Cu-In-Ga-S (CIGS) and Cu-In-Ga-Zn-S (CIGZS) QDs

DDT-capped CIGS QDs can be prepared via the thermal decomposition of CuI, In(OAc)_3_ and Ga(acac)_3_ under an argon atmosphere at 230 °C followed by their shelling with ZnS at 240 °C. The bandgap energy of CIGS QDs increases from 2.15 to 2.60 eV with the increase in the Ga content (In/Ga from 1/0 to 0.7/0.7), which confirms the formation of CuInS-CuGaS solid solutions. CIGS QDs exhibit a deep red emission from 633 to 670 nm, with a relatively modest PL QY of 14%. A marked improvement in PL QY was observed after the ZnS shelling (from ca. 72 to 83% depending on the composition). These QDs were used as a color converter for the fabrication of white-emitting QDs-LEDs [45].

Ga-rich CuIn_1−x_Ga_x_S QDs were prepared by heating CuI, In(OAc)_3_ and Ga(acac)_3_ firstly at 120 °C under argon, then at 250 °C for 3 min and finally for 50 s for the core growth. The shelling of the QDs was carried out by slowly injecting a ZnS solution at 260 °C for 60 min followed by adding OA and holding for 20 min. A surface modification from hydrophobic to hydrophilic was then carried out by adding 3-mercaptopropionic acid followed by heating at 180 °C for 40 min. The obtained CIGS/ZnS QDs exhibit a tunable PL from 546 to 523 nm depending on the In/Ga ratio. PL QYs could reach 70–73%, which is optimal for their application in QDs-LEDs in the form of polymeric films after dispersion into polyvinyl alcohol (PVA) [46]. Kim et al. also reported the preparation of 1-octanethiol (OTT)-capped CIGS/ZnS in ODE at 240 °C with different core compositions by varying the Ga amount. The absolute PL QY of CIGS/ZnS QDs markedly decreases with the decrease in the In/Ga ratio, which originates from the defective surface states in Ga-rich QDs. The performance of CIGS/ZnS was demonstrated in QDs-LEDs [47].

**Figure 6:**
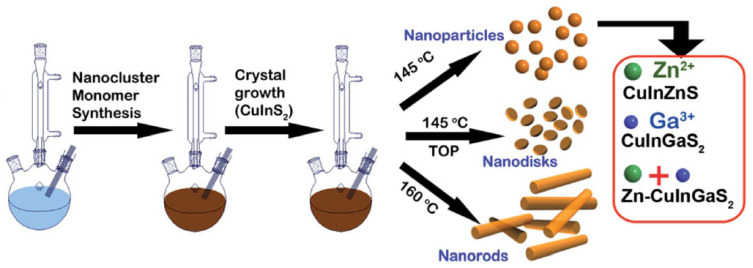
Schematic diagram showing the synthesis of CuInS_2_ QDs, nanodisks and nanorods using CuInS_2_ nanoclusters, and the incorporation of Zn and Ga to synthesize CuInZnS, CuInGaS_2_ and CuInGaZnS_2_ nanoparticles. Used with permission from ref. [48]. Copyright 2015 RSC publishing.

In 2015, a morphology- and composition-controlled synthesis of CuInS_2_ and Zn and/or Ga-doped CuInS_2_ QDs was developed by Perera et al. using (NH_4_)_2_S as a sulfur source [48]. At 145 °C, spherical and nano-disk-shaped crystals were obtained, while nanorods could be produced at 160 °C (Figure 6). A synergistic effect occurred during the incorporation of both Zn and Ga into the core, resulting in an increase in the bandgap energy with an increase in the Ga:Cu ratio. CIGZS QDs prepared with a Ga:Cu ratio of 0.9:0.2 show the highest PL intensity. The exceptional optical properties of CIGZS QDs promote the use of these nanocrystals for applications in PV devices and LEDs.

CIGZS QDs were also produced by the controlled heating of CIGS cores with Zn(stearate)_2_ and Zn(OAc)_2_ in DDT at 240 °C [49]. The results show the formation of a CIGZS solid solution with an ultrabroadband emission. The PL emission spectra can be deconvoluted into three components, with blue emission at 485–500 nm, red emission at 600 nm and NIR emission at 700 nm. CIGZS QDs have excellent quantum efficiency above 80% and were demonstrated to be excellent candidates for the engineering of LEDs.

Concerning QDSSCs, Zhao et al. reported in 2014 the preparation of devices associating TiO_2_, CIGS QDs and the N719 dye (N719: Di-tetrabutylammonium cis-bis(isothiocyanato)bis(2,2′-bipyridyl-4,4′-dicarboxylato)ruthenium(II)) (*vide infra*) [50]. The distribution of the energy levels and the electron-transfer mechanism also show that the FTO/TiO_2_/CIGS/N719 cells block photoexcited electrons transporting from the LUMO level of N719 to the conduction band of TiO_2_, therefore favoring a good charge separation and the optimal injection of electrons from QDs into TiO_2_. Photovoltaic parameters were clearly improved; the *V_oc_* value was increased from 661 to 767 mV for TiO_2_/CIGS and TiO_2_/CIGS/N719, respectively. The *J_sc_* value was also increased from 6.40 to 18.44 mA·cm^−2^ for TiO_2_/CIGS and TiO_2_/CIGS/N719, respectively. Finally, the PCE increased from 2.38% for TiO_2_/CIGS to 7.51% for the TiO_2_/CIGS/N719 device, with an EQE higher than 90%.

### 4.4. Cu-In-Ga-Se (CIGSe) and Cu-In-Ga-Se-S (CIGSSe) QDs

CIGSSe nanocrystals were synthesized by the thermal decomposition of CuCl, InCl_3_, GaCl_3_, sulfur and selenium at 265 °C using OAm as a solvent and capping agent. The bandgap energy could be varied from 0.98 to 2.40 eV by adjusting the In/Ga and the S/Se molar ratios [51]. Due to their high stability, CIGSSe QDs were demonstrated to be good candidates for the fabrication of thin-film solar cells. The cells were prepared by depositing CIGSSe QDs dispersed in toluene on a soda-lime glass substrate coated with molybdenum. After calcination at a high temperature in an Se atmosphere, the sequential deposition of a CdS buffer layer, a transparent ZnO layer and a layer of Al-doped ZnO (Al:ZnO) was conducted. The results show that for an active surface of 0.15 cm^2^ and under AM 1.5 simulated sunlight, the devices have a PCE of 1.02%. The values of *V_oc_* and *J_sc_* are of 0.26 V and 13.96 mA·cm^−^^2^, respectively. These low values are indicative of a recombination loss in the space charge region, likely due to the deterioration of the film, which conducts to trap electrons and holes. The external quantum efficiency (EQE) measured at 800 nm was 38%.

**Figure 7:**
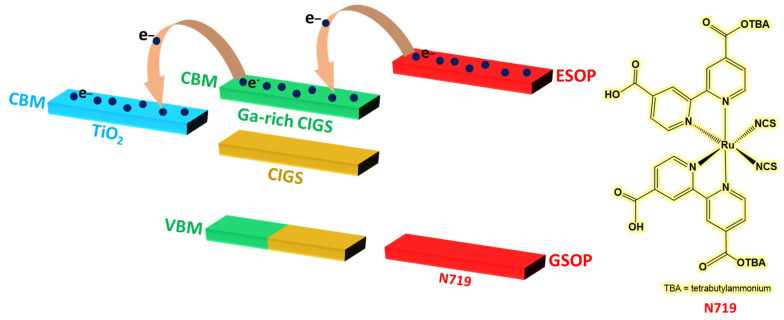
The energy diagram alignment of TiO_2_, Ga-rich CIGS QD, CIGS QD, N719, and iodine-based electrolyte in a DSSC (Adapted from ref. [52]).

In 2012, the solvothermal synthesis route was explored for the synthesis of CIGSe by Lin et al., who stoichiometrically mixed Cu, In, Ga and Se precursors in ethylenediamine and heated the mixture in a sealed autoclave at 200 °C for 48 h [52]. Ga-rich CIGSe nanocrystals exhibit a diameter of ca. 12.9 nm and an *E_g_* of 1.57 eV—a value larger than that of bulk CIGSe (1.19 eV). This increase in the bandgap energy favors the delocalization of photoexcited electrons and is thermodynamically appropriate for the operation and activation of PV cells. The devices were prepared using the sandwich-type cell on an FTO-coated glass substrate, covered by two layers of mesoporous TiO_2_ and a layer of CIGS or Ga-rich CIGS QDs deposited by spin-coating. Then, the electrodes were immersed in a 0.5 mM solution of the N719 Ru-based dye for 24 h. As shown in Figure 7, the level of the conduction band maximum (CBM) of Ga-rich CIGS QDs is −4.11 eV, deeper than that of TiO_2_ (−3.90 eV). Consequently, the photoexcited electrons will migrate from the excited-state oxidation potential (ESOP) level of N719 to the CBM of Ga-rich CIGS, then towards the CBM of TiO_2_, improving the separation of electron/hole pairs, which promotes the better injection of electrons into the cell.

The reference QDSSC prepared using only N719 shows a *J_SC_* of 14.47 mA·cm^−^^2^, *V_oc_* of 751 mV and a PCE of 7.06%. After the introduction of Ga-rich CIGS QDs into the devices, the characteristics are significantly improved and the obtained values are: a *J_SC_* of 15.27 mA·cm^−2^ and a *V_oc_* of 762 mV, and the PCE was increased to 8.02%. The EQE for Ga-rich CIGS/N719 measured at 530 nm was 80.1%.

**Figure 8:**
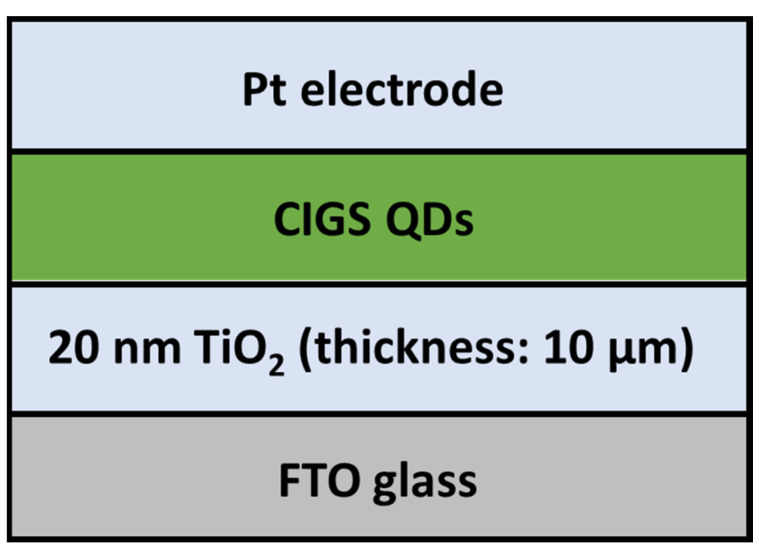
Sandwich-type CIGS-based QDSSC (Adapted from ref. [53]).

In 2015, the solvothermal method was also applied for the synthesis of CIGSe QDs using OAm as a solvent [53]. The as-prepared QDs have an average size between 5 and 10 nm. The calculated bandgap energy value is 2.44 eV, originating from the quantum confinement effect. Sandwich-type FTO/TiO_2_/CIGSe solar cells were developed using these QDs (Figure 8). For the reference sample, the CIGS QDs were replaced by the N719 dye. The reference FTO/TiO_2_/N719 cell showed a PCE of 6.8%, *V_oc_* of 0.76 V and *J_sc_* of 14.3 mA·cm^−^^2^. However, the FTO/TiO_2_/CIGSe cell showed a much lower PCE of 0.057%, with a *V_oc_* of 0.433 V and a *J_sc_* of 0.242 mA·cm^−^^2^. The authors suggest that this significant drop may originate from the bad distribution of CIGSe QDs inside the porous TiO_2_ structure. The as-prepared QDs have an average size of 5 to 10 nm, while the calculated pore size of TiO_2_ was less than 8.8 nm, which can prevent the embedding of QDs inside the porous TiO_2_ film and thus affect the cell efficiency.

**Figure 9:**
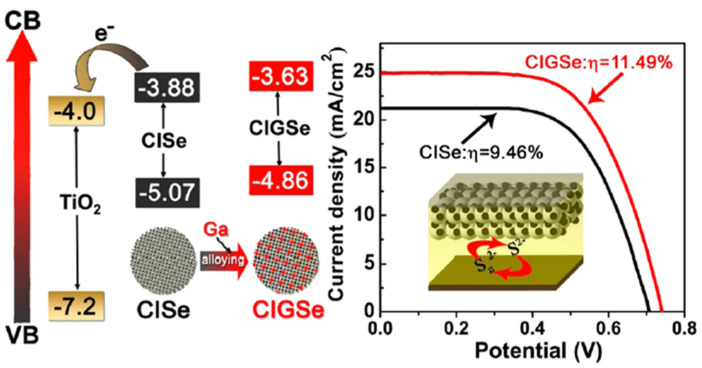
Energy levels of TiO_2_, CISe and CIGSe QDs and current density versus voltage under simulated AM 1.5 sunlight. Used with permission from ref. [54]. Copyright 2017 The American Chemical Society.

In 2017, CIGSe QDs were prepared by the thermal decomposition of CuI, In(OAc)_3_ and Se powder in ODE and OAm. Three Ga precursors were used (GaI_3_, GaCl_3_, and Ga(acac)_3_). The In/Ga molar ratio varied from 1.0:0 to 0.5:0.5. The authors show that the formation of QDs depends on the reaction temperature and demonstrate that nanocrystals could only form at temperatures above 180 °C. The UV-visible absorption edge could be tuned from 950 to 1050 nm by varying the Ga precursor using a fixed Ga/In ratio of 7:3. The PL QY was markedly improved when Ga was incorporated into the CuInSe matrix [54]. This incorporation of Ga into ternary CISe QDs to obtain quaternary CIGSe QDs was demonstrated to be one of the most promising methods for improving the PCE. Solar-cell devices were prepared using the chemical deposition method of CISe or CIGSe QDs on FTO covered with a layer of mesoporous TiO_2_, followed by an overcovering with ZnS and SiO_2_, considered as barrier layers, to suppress the charge recombination of the device (Figure 9) [54]. The optical characterizations show that the *Eg* edge of CIGSe QDs is higher than the *Eg* edges of CISe and TiO_2_, which favors a faster injection rate of photoexcited electrons from the CB of CIGSe into the TiO_2_ matrix. The obtained PV features are excellent for the CIGSe QD-based solar cells, with a PCE of 11.49%, *V_oc_* of 0.740 V and *J_sc_* of 25.01 mA·cm^−2^ against a PCE of 9.46%, *V_oc_* of 0.704 V and *J_sc_* of 21.17 mA·cm^−2^ for CISe QD-based solar cells.

CIGSe QDs can also be synthesized using a microwave-assisted method [55]. The synthesis protocol consists of mixing CuCl, InCl_3_, and GaCl_3_ salts, separately dissolved in trioctylphosphine (TOP) and OAm, and heating for 10 min at a power of 500 W. This is followed by the addition of a solution of Se dissolved in TOP and further heating for 10 min at 600 W. In a second step, a ligand exchange was performed using 3-mercaptopropionic acid. CIGSe QDs exhibit a strong absorption due to the presence of intrinsic defects. The absorption maximum is located at ca. 550 nm, which shows a large blue shift compared to previous reports related to bulk CIGSe and confirms the quantum confinement for the QDs prepared by the microwave-assisted method. The PL emission of CIGSe QDs is located at 650 nm. The prepared CIGSe QDs assembled with reduced graphene oxide (rGO) can be used as counter electrodes in dye-sensitized solar cells. Comparison studies were carried out on TiO_2_/N719 solar cells in the presence of Pt or rGO-CIGSe as counter electrodes in different electrolytes, such as I^−^/I_3_^−^ and thiolate/disulfide (T^−^/T_2_). The photovoltaic performance results showed that the cells prepared with rGO-CIGSe as counter electrodes and the I^−^/I_3_^−^ electrolyte exhibit a *J_sc_* of 8.78 mA·cm^−2^, *V_oc_* of 0.69 V and a PCE of 2.00%. The cells assembled with (T^−^/T_2_) show a *J_sc_* of 7.16 mA·cm^−2^, *V_oc_* of 0.45 V and a PCE of 1.14%. The results are similar when Pt was used as a counter electrode, with a PCE of 3.26% and 1.66% for (I^−^/I_3_^−^) and (T^−^/T_2_), respectively.

Finally, CIGSe nanocrystals with an average diameter of 10–70 nm were prepared by the thermal decomposition of tetrakis(acetonitrile)copper(I) tetrafluoroborate, In(OAc)_3_, Ga(acac)_3_ and diphenyl diselenide in hexadecylamine at 300 °C for 1 h, followed by ligand exchange using EDTA to replace hexadecylamine. CIGSe QDs exhibit a wide absorption in the visible and NIR regions, which makes these QDs of high interest in screen-printing applications [56].

### 4.5. Ag-Ga-In-S (AIGS) QDs

AIGS QDs, with tunable bandgaps, have generated significant interest in several fields of application, such as solar cells or LEDs. AIGS QDs with a pure green emission (ca. 518 nm) and a PL QY of ca. 68% were prepared by two different methods: the first using Ga(DDTC)_3_ (DDTC serving a sulfur source) and the second in the presence of a mixture of Ga(acac)_3_ and 1,3-dimethylthiourea (DMTU), followed by their shelling with GaS at 280 °C for 3 min [57]. The two syntheses were carried out under the same conditions using OAm as a solvent; the temperature of the reactions was set at 150 °C and the In/Ga molar ratio was varied from 1:1 to 0.167:1. Core/shell AIGS/GaS QDs were finally incubated in a chloroform solution containing a small amount of tri-n-butylphosphine (TBP) to control the dispersion of the prepared particles.

**Figure 10:**
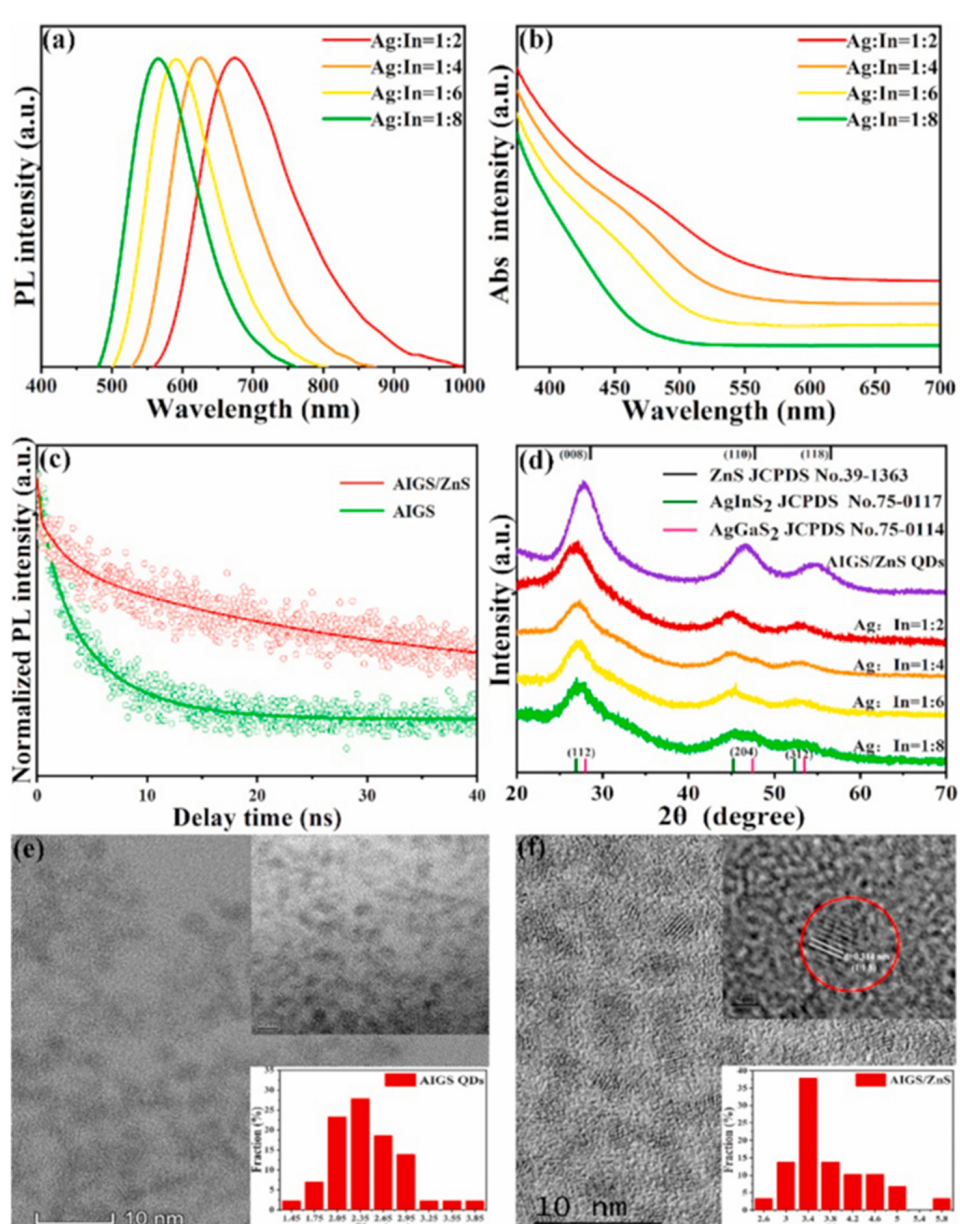
(**a**) PL and (**b**) UV–vis absorption spectra of AIGS/ZnS QDs with different Ag/In ratios; (**c**) Time-resoled PL decay curves of AIGS and AIGS/ZnS QDs; (**d**) XRD patterns of AIGS of different Ag/In ratios and AIGS/ZnS QDs. TEM images of (**e**) AIGS QDs and (**f**) AIGS/ZnS QDs, the insets are the corresponding size distributions. Used with permission from ref. [58]. Copyright 2021 Elsevier.

AIGS QDs used as color converters for LEDs can be prepared by the thermal decomposition of AgNO_3_, In(OAc)_3_ and Ga(OAc)_3_ in ODE, followed by the injection of S at 90 °C. The shelling was carried out by injecting a mixture of Zn(OAc)_2_ and elemental S dissolved in TOP. A series of samples was prepared with different Ag/In ratios, thus allowing us to tune the PL emission wavelength of AIGS QDs between 566 and 674 nm. As expected, a blue shift of the PL emission was observed when decreasing the Ag/In ratio. The average PL lifetime was calculated to be 5.07 ns for AIGS, which confirms the presence of surface and interface-trapped states. After shelling with ZnS, the PL lifetime was increased to 31.35 ns, showing that ZnS strongly influences the surface states of AIGS QDs. XRD results confirm the purity of the obtained AIGS QDs, which crystallize in the tetragonal structure of AgInS_2_ (average diameter of 2.48 nm)m while core/shell AIGS/ZnS QDs crystallize in the blended cubic form of ZnS (average diameter of 3.8 nm) (Figure 10) [58].

The influence on the surface states and defect suppression of AIGS QDs by shelling has been confirmed by the development of AIGS/GaS QDs prepared by the hot-injection method using S, Ag(OAc), In(acac)_3_ and Ga(acac)_3_ as precursors in a mixture of OAm and DDT, followed by their shelling by GaS [59]. The as-prepared QDs exhibit a green PL emission centered at 539 nm and a PL QY of 10.1%.

The high-temperature decomposition method using Ag(OAc), In(OAc)_3_ and Ga(DDTC)_3_ as precursors has been applied for the synthesis of AIGS QDs capped with a GaS shell. The use of an OAm/OA mixture as a ligand allows for an increase in the PL QY from 11.5 to 35.2%, accompanied by a red shift. Post-synthetic modifications of AIGS QDs showed that the addition of ZnCl_2_ (Z-type ligand), which specifically binds to S sites, results in a two-fold increase in the PL QY value (ca. 73.4%). The PL QY was also remarkably improved in the case of core/shell AIGS/ZnS and increased from 9 to 49.5% after treatment with ZnCl_2_ [60].

The effect of transition-metal doping on the optical properties of quinary AgInGaZnS (AIGZS) QDs was recently studied by Galiyeva et al. Mn^2+^-doped AgInGaZnS was prepared through the thermal decomposition of a dithiocarbamate complex of Ag^+^, In^3+^, Ga^3+^, Zn^2+^, and Mn(stearate)_2_ at 220 °C using OAm as a solvent and as a capping ligand. The Mn^2+^ percentage was varied from 1 to 10% relative to the total amount of metal cations, and the molar ratio of Ga/Zn was varied from 0.25 to 2 [15]. The PL QY of Mn:AIGZS was significantly enhanced after Mn doping (from 14.3 to 41.3% for AIGZS and Mn:AIGZS(2.5%), respectively). A large Stokes shift was observed after the doping with Mn^2+^ due to the introduction of surface and interstitial states. Mn:AIGZS QDs show high stability after transfer into water using glutathione tetramethylammonium (GTMA), and no significant decrease in the PL QY was observed (ca. 38.4%) for 2.5% Mn:AIGZS QDs.

**Figure 11:**
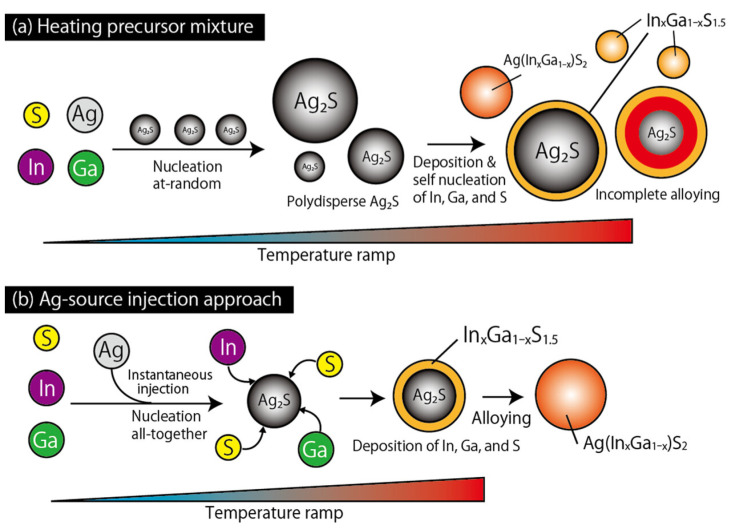
Schematic illustrations of the synthesis of Ag(In_x_Ga_1−x_)S_2_ QDs: (**a**) Heating of the precursor mixture and (**b**) Ag source injection. Used with permission from ref. [61]. Copyright 2023 The authors.

The organic-phase synthesis of quaternary AgIn_x_Ga_1−x_S_2_ QDs via the thermal decomposition of the silver precursor in the presence of the indium and gallium precursors usually results in a marked precipitation of Cu_2_S or Ag_2_S. Recently, a new approach has been developed consisting of the hot injection of the Ag precursor into a mixture solution of dithiocarbamate complexes of In^3+^ and Ga^3+^ (Figure 11). After shelling with GaS, the as-prepared AgIn_x_Ga_1−x_S_2_ QDs show a strong green emission with a tunable PL emission between 499 and 543 nm, indicating their potential application in the field of light-emitting diodes (LEDs) [61].

AIGS QDs can also be prepared in aqueous media using the microwave-assisted route using AgCl, InCl_3_ and Ga(NO_3_)_3_ as precursors and glutathione and citric acid as surface ligands. The QDs obtained after multiple rounds of ZnS shelling show a high-PL QY of 79%. These QDs, with excellent photoluminescence properties, are good candidates for manufacturing LEDs after their incorporation into polyacrylamide/polyvinyl alcohol (PAAm/PVA) hydrogels [62]. The white-light-emitting LED exhibits a high color-rendering index of 92.1 and a correlated color temperature of 3022 K.

### 4.6. Ag-Ga-S(Se) (AGS(Se)) and Ag-Ga-Zn-S(Se) (AGZS(Se)) QDs

Due to the interest in preparing highly luminous and ultra-stable Cd-free QDs with wide-tunable short-emission wavelengths for displays and lighting devices, a series of Ag-doped Ga-Zn-S QDs was developed by using a one-pot non-injection process. The key parameter affecting the PL emission was determined to be the Zn/Ga molar ratio, which allows us to tune the bandgap of the host and subsequently the luminescent characteristics of the Ag^+^ dopant ions. The emission of Ag ions was found to emanate from the recombination of electrons located within the CB of the alloyed QDs and holes in the energy level of the Ag dopant. The obtained QDs show excellent color tunability while covering the complete spectrum from violet to aqua, spanning from 370 to 540 nm. An average particle size of 2.4 nm for the Ag:Zn-Ga-S core and of 10.1 nm for the Ag:Zn-Ga-S/ZnS core/shell QDs with a cubic zinc-blended structure was determined [63]. This study further reports high-PL QYs up to 85% for as-synthesized type-I Ag:Zn-Ga-S/ZnS core/shell QDs. Noteworthily, the high-PL QY of the prepared QDs was found to be stable at an annealing temperature of up to 300 °C under continuous UV irradiation for 24 h, but also after the transfer of the native oil soluble QDs into aqueous media by ligand exchange using 11-mercaptoundecanoic acid.

A similar non-injection method was used for the synthesis of Ag-Ga-S (AGS) QDs and their quaternary Ag-Ga-Zn-S (AGZS) derivatives by alloying with Zn^2+^ ions [64]. For the synthesis of ternary AGS QDs, a mixture of AgI, Ga(acac)_3_, and S in a DDT/OAm solution was used. The Ag/Ga molar ratio was kept below 1 as the scarcity of Ag relative to Ga plays a crucial role in achieving a high-PL QY, a common feature for I-III-VI_2_-type QDs. An optimal Ag/Ga molar ratio of 1/8 was determined through experimentation. For the synthesis of quaternary AGZS QDs, a similar method to that for ternary QDs was adopted, simply by adding ZnCl_2_ to the reaction mixture. The core QDs were shelled by ZnS due to a weak lattice mismatch between AGS and ZnS, which allows us to form core/shell AGS/ZnS and then AGZS alloyed QDs. In regard to the correlation between QD composition and bandgap variation, the resultant AGS/ZnS and AGZS/ZnS QDs demonstrated a well-ordered progression of PL colors, ranging from blue (450 nm) to green (525 nm), while maintaining a high-PL QY of up to 66% after ZnS shelling. The *E_g_* values of the QDs are in the range of 2.90 to 3.10 eV with increasing the Zn^2+^ content. The average sizes of both core and core/shell QDs fell within the ranges of 2.6–2.8 nm and 5.0–5.3 nm, respectively. Some variations in size were observed, influenced by the composition of the QDs. This method specifically leverages the extensive compositional flexibility unique to I-III-VI_2_ QDs, which surpasses that of II-VI and III-V nanocrystals. This versatility arises from the ability to expand their composition into quaternary solid solutions through isovalent ion substitution or alloying with the ZnS phase.

Other groups have also reported relatively fast preparation methods to access high-quality AGS and AGZS nanocrystals. One such investigation delves into a colloidal hot-injection method for producing AGS nanocrystals, utilizing a reactive sulfur precursor pre-formed by dissolving elemental S in a mixture of OAm and DDT. This methodology was further expanded to prepare alloyed AGZS core/shell QDs through a single-step fast process that avoids the purification of the AGS core [65]. Both AGS and AGZS QDs are spherical and show uniformity in size (ca. 5.1 nm) and a high degree of crystallinity (tetragonal for AGS and cubic zinc-blended for AGZS QDs due to the increase in Zn^2+^ ion content). The presence of the amorphous ZnS shell was found to significantly enhance PL QYs (up to 21.2%) mainly due to the use of reactive S and Zn^2+^ precursors at high reaction temperatures. By tuning the non-stoichiometry of AGS, the bandgap energy *E_g_* of the QDs could be adjusted from 2.83 to 2.98 eV by the increase in the Ga content. This resulted in tunable PL colors, ranging from aqua (490 nm) to blue (461 nm). The study showcases the one-pot synthesis of AGZS QDs, along with their capacity for bandgap engineering. This kind of advancement is vital for potential industrial scalability and for tailoring the optical properties of I-III-VI_2_ materials.

Xiulin et al. described another synthesis of Ag-Ga-Zn-S NCs via a facile one-pot method to develop narrow-bandwidth blue-emitting I-III-VI_2_ QDs, allowing for higher color purity than the earlier-reported AGZS/ZnS NCs (emitting blue light at 450 nm with a full-width at half-maximum (fwhm) beyond 80 nm) [66]. The prepared AGZS QDs emit blue light at 470 nm and show an improved fwhm value of 48 nm. Furthermore, the bandwidth and the PL QY of alloyed AGZS QDs displayed a significant reliance on the Ag/Ga and Ag/Zn ratios. Notably, a high-PL QY of 16.7% and an fwhm narrower than 50 nm were achieved when utilizing an Ag/Zn feeding ratio of 4:1 and an Ag/Ga feeding ratio of 1:8. Subsequently, these high-quality AGZS QDs with tightly confined emission spectra were effectively integrated into solution-processed QLEDs. The obtained AGZS NCs show a regular circular morphology with an average size of 4.2 nm and a tetragonal structure, confirming the formation of high-quality AGZS QDs. Moreover, by adjusting the Ag/Zn and Ag/Ga ratios in the synthesis process, the study further discusses the underlying mechanism behind the narrow bandwidth emission, which predominantly arises from the enhanced radiative recombination process between the CB and Ag^+^ vacancy. This study not only lays the foundation for the controlled production of narrow-bandwidth I−III−VI_2_ QDs using a single-step approach but also opens promising avenues for future applications within the display industry.

To further improve the quality and the PL intensity of AgGaS/ZnS QDs, Lu et al. developed a two-step method to synthesize core/shell/shell AGS/ZnS/ZnS QDs [67]. The internal ZnS shell was created using a one-pot method, while the outer ZnS shell was incorporated through the gradual injection of a Zn precursor. The AGS/ZnS/ZnS QDs exhibit a PL emission at 520 nm, with the highest recorded PL QY of 96.4% for Ag and Ga containing I-III-VI_2_ QDs. AGS/ZnS/ZnS QDs also exhibit a narrow size distribution, with a mean diameter of 6.5 nm. The increase in the Zn^2+^ amount for the outer ZnS shell deposition induced a shift in the XRD peaks towards higher angles, converging towards the ZnS phase. This shift served as additional evidence for the formation of a core/shell structure. Furthermore, as successive rounds of Zn-stock injection were employed, the XRD peaks became narrower, indicating the progressive enlargement of the ZnS shell. Likewise, after three successful batches of Zn-stock injection, the average lifetime of AGS/ZnS/ZnS QDs increased from 132.40 to 1722.90 ns, with a gradual decrease in their bandgap from 2.93 to 2.85 eV, respectively. The inner shell aided in reducing the lattice mismatch between the QDs and the outer ZnS shell, thus facilitating the growth of a thicker exterior shell. The study also investigated the impact of shelling temperatures and halogen ions present in the Zn precursors on the shelling process and the structure of the core/shell arrangement.

**Figure 12:**
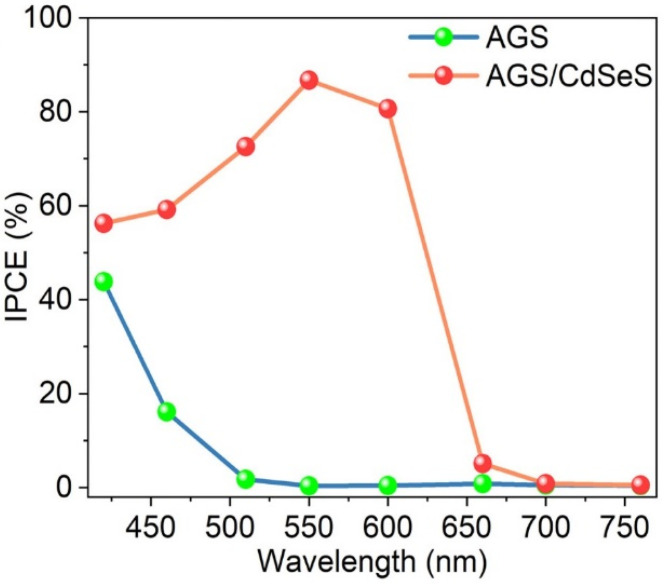
IPCE spectra of AGS core and AGS/CdSeS core/shell QDs-based PEC cells. Used with permission from ref. [68]. Copyright 2021 Elsevier.

A two-step hot-injection method was described for the growth of core/shell AgGaS_2_/CdSeS QDs [68]. Near-spherical-shaped QDs with a zinc-blended structure were obtained. The bare AgGaS_2_ core QDs (*E_g_* = 2.75 eV) have a limited light-absorption range up to 450 nm due to their large bandgap, and the PL emission is located at 536 nm. After CdSeS shelling, AgGaS_2_/CdSeS QDs (*E_g_* = 1.97 eV) display an extended absorption up to 650 nm. These core/shell QDs also exhibit a red-shifted PL emission peak at 710 nm with an unusually long exciton lifetime of 1.9 μs. The bare AgGaS_2_ core QDs revealed a quasi-spherical shape with an average size of 3.2 nm and a tetragonal chalcopyrite structure. After the CdSeS shell growth, the size of AgGaS_2_/CdSeS QDs increases to 5.1 nm and the interplanar spacing matches well with the zinc-blended phase of CdSeS, which confirms the deposition of CdSeS on the AgGaS_2_ core. These characteristics indicated a type-II band alignment, facilitating the spatial separation of photogenerated electron-hole pairs, which is of high interest for various QD-based devices. This was demonstrated by the use of AgGaS_2_/CdSeS QDs in the engineering of optoelectronic devices, including a solar-driven photoelectrochemical (PEC) cell and a photodetector. The performance of the QD-sensitized devices represents a significant advancement towards expanding the optical responsiveness and customizing the band structure of wide-bandgap QDs for effective optoelectronic applications. The improvements described in this study encompass the development of interfacial gradient alloyed CdSe_1−x_S_x_ QDs to enhance charge dynamics and the alteration of the shell composition (such as AgInS/Se) to achieve broader near-infrared absorption, beneficial for sustainable optoelectronic applications. The authors investigated AgGaS_2_/CdSeS core/shell QDs as sensitizers for QDSSCs, with AGS/CdSeS QDs exhibiting enhanced light absorption in the visible region. The fabrication of the cell was finalized by coating the photoanode surface with two ZnS layers to limit photocorrosion. During the operation, the device under standard sunlight conditions (AM 1.5 G, with an intensity of 100 mW cm^−2^) generated electron-hole pairs in the type-II band-structure QDs. These pairs consisted of photoexcited holes, which were promptly consumed by sacrificial reagents within the electrolyte, while the photogenerated electrons transferred from QDs to TiO_2_ and ultimately reached the surface of the Pt counter electrode. The fabricated photoanode of AGS-bare core QDs exhibited a photocurrent density of around 2.6 mA·cm^−2^. However, upon introducing core/shell AGS/CdSeS QDs onto the photoanode, the photocurrent density significantly increased to approximately 6.8 mA·cm^−2^. These values were notably higher than that achieved with the bare TiO_2_ photoelectrode, which yielded approximately 0.3 mA·cm^−2^, further confirming QDs as the predominant source of the generated photocurrent. Further, to assess the performance of the QD-based device in the visible light range, a 420 nm cut-off filter was used. Under these conditions, the AGS-based photoelectrode showed a very low photocurrent density of 0.4 mA·cm^−2^, due to the limited absorption of visible light by AGS QDs. In contrast, the AGS/CdSeS QDs-based photoanode produced a significantly higher photocurrent density of 4.8 mA·cm^−2^—a value approximately twelve times higher than that of the AGS-bare QDs. The IPCE values for PEC cells using AGS QDs showed a clear decline, starting at around 43.8% at a 420 nm wavelength and dropping to approximately 1.7% at 510 nm. Conversely, the PEC device utilizing AGS/CdSeS QDs exhibited significantly improved photon-to-electron conversion efficiencies across the visible spectrum, spanning from 420 nm to 700 nm. It reached a remarkable 86% at 550 nm, whereas the efficiency for AGS-bare QDs at this wavelength was close to 0 (Figure 12). However, even if the deposit of a CdSeS shell at the surface of AGS QDs was demonstrated to be effective in improving the PCE, the toxicity of Cd will hinder the further application of this strategy for large-scale production.

**Figure 13:**
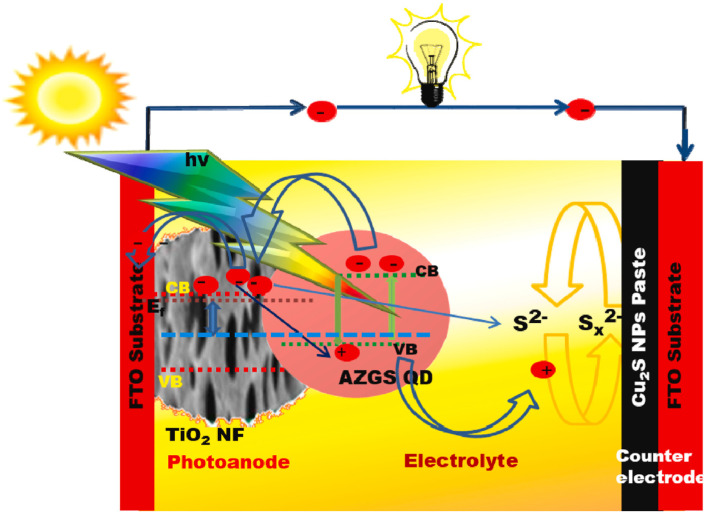
Schematic representation of the fabricated QDSSC. Used with permission from ref. [69]. Copyright 2022 Elsevier.

By using the colloidal hot-injection method, Kottayi et al. incorporated Zn into the Ag-Ga-S host matrix to develop quaternary Ag-Ga-Zn-S QDs with a narrow bandgap (2.10 eV), fast electron-transfer ability (average lifetime of 67.75 ns), a wide absorption range, and a narrow PL emission in the NIR region [69]. The PL emission of Ag-Ga-Zn-S QDs is a symmetrical single peak with a maximum intensity at 710 nm. The fwhm is 25 nm, indicating that QDs exhibit a narrow size distribution and that these crystals are defect-free. The QDs exhibit an orthorhombic crystal structure with an average diameter of 6.9 nm. An AgZnGaS_3_-based solar cell was fabricated using the AGZS/TiO_2_ photoanode and subjected to ac-impedance and photovoltaic performance tests. The optical measurements on QDs indicate a low bandgap (2.1 eV) and absorption onset towards the NIR region, which could be helpful for achieving high light-harvesting ability with an increased Fermi energy level (E_f_) and an upward shift in the CB of AGZS QDs. The disparity in energy levels heightens the impetus for electron transfer, enabling electrons to move efficiently from the CB of AZGS QDs to the CB of TiO_2_ nanofibers (NFs). This process facilitates both the effective extraction of photoelectrons and the swift transfer of electrons from QDs to TiO_2_ NFs. The accumulated holes within the AGZS QDs were subsequently consumed by the redox active electrolyte. The oxidized electrolyte (polysulfide, S_x_^2−^) was converted back to sulfide (S^2−^) by electrons at the Cu_2_S counter electrode (Figure 13). As a result, the PCE of the AgZnGaS_3_ QDs-based QDSSC showed a significant improvement compared to previously reported sulfide-based QDSSCs. The photovoltaic performance was assessed through the *J-V* curve, revealing key parameters: *J_sc_* = 12.31 mA·cm^−2^, *V_oc_* = 0.51 V, and *FF* = 0.62. The notably high *J_sc_* value is primarily attributed to the excellent optical characteristics and electron chemical reactivity of the AZGS/TiO_2_-based photoanode. As a result, the estimated PCE of the QDSSC was recorded at 3.81%. A crucial parameter influencing photoanode performance is the charge-transfer resistance, denoted as R_ct_ and represented by the diameter of the low-frequency right semicircle. The Nyquist plot revealed that the AZGS/TiO_2_ photoanode possessed a notably low R_ct_ value (45.21 Ω). These findings indicate that the AZGS/TiO_2_ cell exhibits exceptional electron-transfer capabilities, thereby enhancing the performance of the fabricated QDSSC.

The same group slightly modified the colloidal hot-injection method by using an S and Se stock solution in DDT/OA instead of S to synthesize quinary Ag-Zn-Ga-S-Se alloyed QDs. The as-prepared QDs show a wide absorption range in the NIR region with an *E_g_* of 1.37 eV, which confirms that these QDs are effective sensitizers [70]. A single PL emission peak from 830 to 880 nm with an fwhm of 20 nm was observed. An improved electron-transfer ability with an average lifetime of 42.64 ns was reported. AGZSSe QDs were used as sensitizers to fabricate AGZSSe QDs/TiO_2_ NFs as photoanodes for based QDSSCs. As expected, the wide-range light-harvesting ability and the enhanced electron transfer from the CB of AGZSSe QDs to TiO_2_ NFs resulted in a greater PCE than those of earlier reported similar QDs (AIS, AIZS, AIZSe). The corresponding photovoltaic parameters *J_sc_*, *V_oc_*, and *FF* were recorded at 14.20 mA·cm^−2^, 0.54 V, and 0.64, respectively, and were obtained from the *J-V* curve of the fabricated AGZSSe/TiO_2_ photoanode-based QDSCs. The PCE was calculated to be 4.91%. The recorded value for R_ct_ was found to be 26.78 Ω, indicating efficient electron transportation, contributing to the improved performance of the QDSSC.

Apart from the well-elaborated high-temperature approaches utilizing heating-up and hot-injection synthetic protocols, mild-temperature aqueous-synthesis methodologies are of particular interest for developing water-dispersible QDs that are readily applicable in biology and medical-related fields. However, the aqueous-synthesis protocol appears to be less developed for the synthesis of I-III-VI_2_-type QDs and requires more effort to prepare high-quality quaternary derivatives. A mild colloidal aqueous-phase synthesis of ternary Ag-Ga-S QDs using glutathione as a capping ligand was described (synthesis conducted at a temperature below 100 °C) [71]. The average size of the QDs obtained is ca. 2 nm, as determined by atomic force microscopy (AFM). Size-selective sorting was conducted through repetitive centrifugation and the addition of a non-solvent. The QD chemical composition was nearly stoichiometric for fractions containing larger nanocrystals. However, a discernible trend towards the shortage of silver and the excess of sulfur emerged with decreasing QD size. The absorption edge of the prepared Ag-Ga-S QDs also shifts towards higher energies when the size of the nanocrystals decreases. The obtained Ag-Ga-S QDs likely exist in a metastable state, potentially in orthorhombic, rhombohedral, or rocksalt-type phases (presumably due to substantial internal pressure within the crystallites). The Ag-Ga-S QDs display a narrow indirect bandgap, in contrast to the bulk tetragonal AgGaS_2_. A broadband PL emission is observed only for medium-sized fractions, for which the energy of the PL emission maximum increases with decreasing QD size, and the intensity of PL clearly demonstrates a nonmonotonic dependence on size. The Raman spectra of the Ag-Ga-S QDs were compared to those of bulk AgGaS_2_ crystals, as well as to those of Ag-In-S QDs synthesized using a similar approach. An analysis of the features in the Raman spectra of the synthesized Ag-Ga-S QDs indicates that the positions and widths of the Raman bands observed are strongly influenced by the contribution of surface phonons, stemming from the high surface-to-volume ratio. Finally, optical absorption spectra collected after three months of storing demonstrate that the size-selected Ag-Ga-S QDs solutions are stable.

### 4.7. Ag-In-Ga-Se (AIGSe) QDs

GaS-shelled AIGSe QDs with NIR emission, and thus of high added value for in-vivo bio-imaging applications, were prepared via the high-temperature decomposition of Ag(OAc), In(acac)_3_, and Ga(acac)_3_ as metal precursors and an appropriate amount of selenourea as selenium source in a mixture of OAm and DDT (10 min at 150 °C and 10 min at 300 °C) [72]. The Ag/(In + Ga) ratio was set at 0.67 and the In/(In + Ga) ratio was varied from 1 to 10. The study of the optical properties shows that the shelling with GaS allows us to minimize surface defects and improves the PL QY value. The optimal PL QY was obtained for AIGS/GaS QDs prepared with an In/(In + Ga) molar ratio of 0.75 (ca. 14%). The study also shows that the remaining surface defects are due to the partial covering of AIGS QDs by the GaS shell.

A two-step heat treatment was used for the synthesis of quinary AgInGaSSe (AIGSSe) QDs. The synthesis protocol involves mixing Ag(OAc), In(acac)_3_ and Ga(acac)_3_ as metal precursors. Thiourea and selenourea were used as chalcogen precursors for S^2−^ and Se^2−^. Both metal and chalcogen precursors were dissolved in a mixture of OAm and DDT followed first by heating at 100 °C for 30 min and then at 250 °C for a further 30 min. The AIGSSe QDs were then coated by GaS shells at 300 °C for 15 min. During the synthesis of AIGSSe QDs, a red shift of the PL emission was observed from 580 to 790 nm. After covering with the GaS shell, the PL QY was improved, with the highest value (50%) obtained for the QDs emitting at 580 nm. The AIGSSe QDs prepared with the Se/(S + Se) molar ratio of 0.5 present an emission peak located at 790 nm, which is favorable for bio-imaging applications [73].

### 4.8. Cu-In-Se-S (CISeS) and Cu-In-Zn-Se-S (CIZSeS)QDs

**Figure 14:**
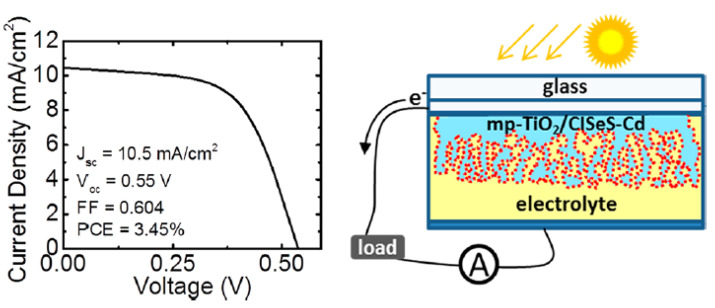
Current density versus voltage under simulated AM 1.5 sunlight for QDSSC fabricated using QDs treated with Cd-oleate at 50 °C and incorporating a scattering layer (**left**). Schematic of the QDSSC (**right**). Used with permission from ref. [74]. Copyright 2013 The ACS Publishing.

CISeS QDs with a narrow bandgap of up to 0.5 eV were prepared at 230 °C by the hot-injection method using CuI and In(OAc)_3_ as precursors, OAm as a ligand and DDT as a sulfur source. The alloyed QDs were obtained by slowly injecting a TOP-Se solution at 230 °C [74]. A cation exchange was then carried out with Cd, using a Cd-oleate solution at two different temperatures (50 and 125 °C), to vary the cation exchange percentage. After this exchange, the CISeS-Cd QDs were purified and finally recapped with tert-butylamine (tBA). The authors showed that the bandgap energy is considerably reduced with the increase in Se content. The PL QY was drastically increased for CISeS-Cd QDs and reached values above 80% for QDs with a high selenium content (50-fold more than CISeS). To improve the photovoltaic performance of CISeS QDs, partial cation exchange with Cd was demonstrated to be an option of interest. This procedure allows us to reduce recombination losses through the encapsulation of QDs in a thin Cd(Se,S) passivating shell, leading to reduced surface trapping. The PV cells were prepared by soaking mesoporous TiO_2_ fixed on an FTO slide prepared by repeated, automated screen printing in a QDs/octane solution for 24 h (Figure 14). The best results were obtained for the cell prepared using Cd-oleate treated QDs at 50 °C with a PCE of 3.45%, a *V_oc_* of 0.55 V and a *J_sc_* of 10.5 mA·cm^−^^2^.

The same authors studied the effect of the injected amount of Se and of the temperature on the optical properties of CISeS QDs [75]. The synthesis of the QDs was conducted under similar experimental conditions except that the Se precursor was dissolved in a mixture of OAm/DDT instead of TOP. The results indicated that the optical properties depend on the composition of the QDs. The increase in the Se content favors a red shift, while the temperature has little influence.

Quinary alloyed CIZSeS QDs can easily be prepared using the hot-injection method [76]. The decrease in the S/Se molar ratio from 9/1 to 5/5 allows us to shift the absorption onset from 820 to 930 nm, indicating a decrease in the energy bandgap and a broadening of the light absorption range. Simultaneously, the PL emission peak was shifted from 790 to 820 nm. PL lifetime measurements indicate that the charge recombination loss is reduced in CIZSeS compared to CIZSe QDs due to the decrease in surface-defect states acting as non-recombination centers. Cation (Zn) and anion (Se) co-alloying was demonstrated to be a powerful method to improve the PV performance of CIS QDs. An adequate S/Se ratio allows us to optimize *J_SC_*, *V_OC_* and FF values compared to ZCIS and ZCISe QDs. Using Zn_0.__4_Cu_0.__7_In_1.__0_S_x_Se_2−x_ (S/Se ratio of 6/4) QDs, an exceptional PCE value of 14.48% (certified efficiency of 14.4%) was obtained. The high performance observed was demonstrated to be linked to the inhibition of the interfacial charge recombination process in QDSSCs when using CIZSeS compared to CIZSe QDs.

The same authors later demonstrated that the performance of the QDSSCs could further be improved by a QD secondary deposition approach via the creation of a metal (Mg^2+^, Ti^4+^, Ca^2+^ or Sr^2+^) oxyhydroxide layer around QD-pre-sensitized photoanodes [77]. This strategy allows us to improve the loading in QDs at the surface of the photoanode (by 38% using Mg^2+^) due to the creation of new adsorption sites. The average PCE of the CIZSeS-sensitized QDSSC was increased to 15.31% (certified PCE of 15.20%) (*J_SC_* = 26.52 mA·cm^−2^, *V_OC_* = 0.802 V and *FF* = 0.720), which is the highest value recorded to date for liquid-junction QDSSCs.

Very recently, a continuous flow reactor synthesis was developed to produce high-quality and gram-scale (up to 3.5 g) Cu-In-S, Cu-In-Se and Cu-In-Zn-Se-S QDs using CuI, In(OAc)_3_, Zn(OAc)_2_ as metal precursors, Se-diphenylphosphine (DPP) as an Se precursor and OAm as a ligand [78]. The PV performance of CIZSeS QDs was also demonstrated to be dependent on the capping ligand of the dots [78]. The replacement of the OAm ligand by S^2−^ allows us to improve electron transfer and thus PV parameters like *V_OC_*, *J_SC_* and FF in a PV cell in which the TiO_2_ film functions as a n-type semiconductor. The PCE was increased by ca. 25% using S^2−^-capped CIZSeS QDs compared to OAm-capped QDs.

### 4.9. Ag-Cu-In-S (ACIS) QDs

A mild aqueous synthesis of colloidal ACIS QDs (2–4 nm sized) using the glutathione (GSH) ligand followed by ZnS shelling was developed [79]. These QDs were found to behave as solid solutions rather than mixtures of AIS and CIS phases, as demonstrated by Raman spectroscopy. The XRD patterns of the core QDs exhibit three markedly broadened reflections, which were indicative of the tetragonal chalcopyrite-like structure. As Cu was incorporated, the observed reflections show a gradual shift towards smaller values. These shifts were quite similar in core/shell ACIS/ZnS QDs due to the incorporation of Zn^2+^ ions into the QD core, which caused the lattice contraction. The bandgap and energy levels of the synthesized ACIS/ZnS QDs revealed a non-monotonic pattern, where the bandgap decreased from AIS (2.15 eV) to ACIS QDs (1.80 eV, 50 mol% Cu) before re-increasing for Cu-richer ACIS compositions and pure CIS QDs (1.98 eV). This was demonstrated to originate from a band-bowing effect. In comparison to the ACIS core QDs, the core/shell ACIS/ZnS QDs displayed band edges shifted towards shorter wavelengths with slightly higher bandgap values. This shift in band edges and increase in bandgap values was attributed to the incorporation of Zn^2+^ ions within the ACIS cores. Similarly, Stroyuk et al., while attempting to synthesize quinary Ag_x_Cu_1−x_InS_y_Se_1−x_ (ACISSe) QDs by spontaneously alloying aqueous GSH-capped AIS, CIS, AISe, and CISe QDs in aqueous solutions, reported evidence of a band-bowing effect. The PL peak energies of QDs with both Cu and Ag were observed to be lower than those of QDs composed solely of Cu or Ag. At the same time, the QDs with Cu and Ag exhibit longer average PL lifetimes in comparison to QDs containing only Ag or Cu [80].

To further develop environmentally friendly ACIS QDs, an aqueous colloidal solution approach was utilized to prepare Cu-doped AIS and ACIS/ZnS QDs followed by post-synthetic size selection. The ZnS-shelled QDs showed a PL QY of 15% with an almost similar PL band position. The study further discusses the PL quenching and the red shift in the PL band maximum from 630 to 780 nm due to the Cu-doping of non-stoichiometric AIS QDs. Size selection was demonstrated to be an effective method for influencing both the spectral PL characteristics and the PL efficiency, yielding ACIS/ZnS QDs with PL QYs reaching nearly 60%. Moreover, 2-Isopropanol was used as a non-solvent to obtain nine fractions of synthesized QDs revealing different optical properties. The variation was evidenced by the difference in average sizes (from around 3 to 2 nm and smaller). This decrease in the average size yielded a blue shift of the PL emission. A series of emission colors varying from deep-red to bluish-green, with PL QYs increasing from 11% for the first fraction to up to 58% for the smallest Cu-doped AIS/ZnS QDs fraction, was observed [81].

### 4.10. Ag-Cu-In-Se (ACISe) QDs

**Figure 15:**
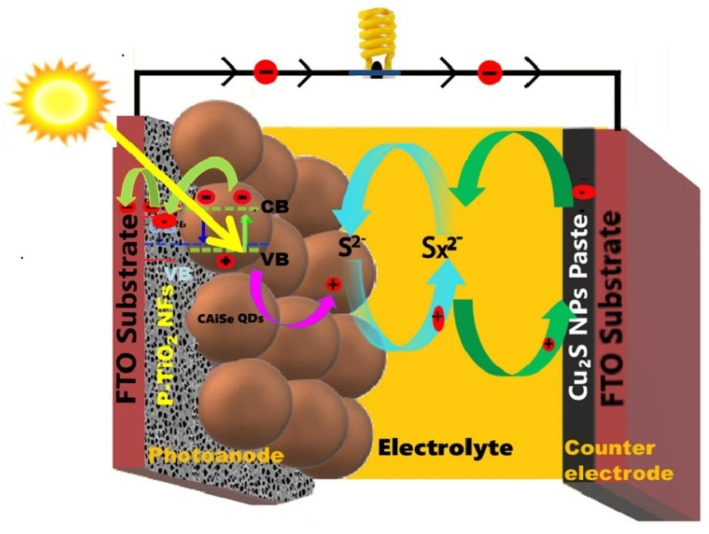
Schematic representation of the fabricated QDSC. Used with permission from ref. [82]. Copyright 2020 Elsevier.

Quaternary ACISe QDs can be prepared via a two-step hot-injection method [82]. The obtained Cu_2_AgInSe_4_ QDs exhibit an average crystallite size of 4.8 nm with a tetragonal (kesterite phase) structure, and the Tauc plot shows a bandgap of 1.93 eV. The developed QDs show evenly shaped PL peaks in the 800–950 range with a maximum PL emission intensity at 845 nm. The good optical properties of synthesized QDs allowed for the fabrication of Cu_2_AgInSe_4_ QDs-sensitized porous TiO_2_ NFs as photoanodes for QDSSCs. Cu_2_AgInSe_4_ QDs-sensitized solar cells were fabricated due to their broad light-harvesting ranging from the UV-visible to the IR region by depositing ACISe QDs over porous-TiO_2_ NFs to form the photoanode (Figure 15). The *J-V* characteristics of the developed QDSSCs were examined. The resultant ACISe QDs/p-TiO_2_ NF-based QDSSC exhibited a PCE of 4.24%, which is higher than values reported for binary and ternary QDs. The good performance of the developed QDSSC was attributed to the high loading capacity of QDs onto p-TiO_2_ NFs; the significant photon harvesting capability of ACISe QDs towards the NIR region; and appropriate band alignment at the interface of TiO_2_ NFs. The absorption onset of QDs towards the NIR region causes a rise in the *E_f_* level and thus an upward shift in the CB of AICSe QDs. This CB upshift, in a cascading effect, serves as the driving force for the transfer of electrons from the CB of AICSe QDs to the CB of TiO_2_ NFs. As a result, an increase in both current density and the open-circuit voltage could be observed. The electrochemical impedance studies were conducted by analyzing the Nyquist plot of AICSe/p-TiO_2_ NFs-based QDSSCs. The recorded R_ct_ value was 20.9 Ω, indicating a charge-transport resistance at the junction between the photoanode and the electrolyte. This resistance was indicative of the effective prevention of electron recombination between ACISe QDs and the electrolyte. This effect could be attributed to the high QD absorption capacity on the porous TiO_2_ NFs, which facilitates the seamless transport of electrons without encountering any obstacles.

### 4.11. Ag-Cu-Ga-Se (ACGSe) QDs

**Figure 16:**
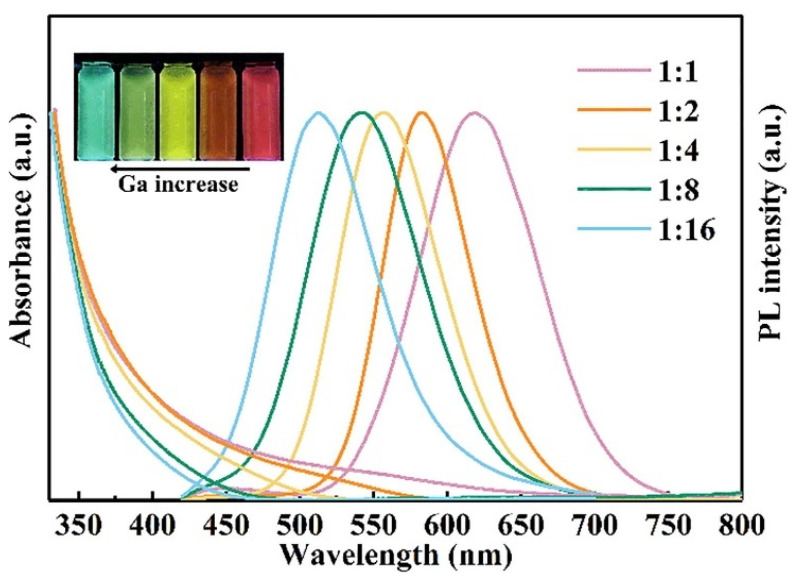
UV–vis absorption and PL spectra of the CAGSe/ZnSe QDs synthesized under a series of (Ag + Cu)/Ga ratios with the ratio of Ag/Cu fixed at 3/7. Inset: digital photographs of samples irradiated under the 365 nm UV lamp. Used with permission from ref. [83]. Copyright 2021 Elsevier.

To date, there has been limited documentation produced on quaternary tunable I-III-VI_2_ QDs with monovalent substitutions of group IB (Ag, Cu). A facile one-pot method was developed to prepare quaternary core/shell Ag-Cu-Ga-Se/ZnSe QDs [83]. A high-PL QY of 71.9% and a color-tunable emission ranging from 510 to 620 nm were accomplished via adjustments of OAm dosages and precursor ratios [Ag/Cu and (Ag + Cu)/Ga] (Figure 16). The average size of the ACGSe/ZnSe QDs ranged from 4.4 to 5.7 nm with the increase in OAm dosage, and the dots exhibit a chalcopyrite structure. Due to their exceptional optical characteristics, ACGSe/ZnSe QDs were utilized in the fabrication of white-light-emitting LEDs. These outcomes highlight the promising prospects of these ACGSe-based quaternary QDs, which are highly suitable options for various applications [83].

### 4.12. Cu-Ga-S(Se) and Cu-Ga-Zn-S(Se) QDs

Cu-Ga-S (CGS) has a much higher bandgap (2.43 eV) than conventional I-III-VI_2_ semiconductors such as AgInS_2_ or CuInS_2_. CGS nanocrystals and Cu-Ga-Zn-S (CGZS) QDs were initially developed to gain access to non-Cd materials emitting in the blue region that could be used as emitting components for LEDs. Indeed, a high degree of Cu deficiency relative to Ga cations affords QDs a wider bandgap due to the lowering of the VB maximum as the repulsion between Cu d and S p orbitals is attenuated. Cu-deficient CGS QDs were first prepared via the “non-injection” method from CuI, GaI_3_ and S using DDT and OAm as the capping ligand and solvent, respectively, followed by ZnS shelling and alloying [84]. The high-energy emission component (at ca. 485 nm) likely originates from the radiative recombination of an electron in the CB with a hole trapped in the Cu vacancy, while the weaker energy tail emission may be assigned to DAP recombination. The obtained CGZS QDs exhibit high-PL QYs (78–83%).

The doping of these QDs with Mn^2+^ ions was later investigated [85]. Mn was introduced after the CGZS QDs growth, and a double ZnS shelling was then conducted, allowing Mn^2+^ to diffuse into the CGZS lattice. The obtained QDs exhibit two emissions: the blue emission of the CGZS host associated with the Mn^2+^-related emission at ca. 595 nm. By varying the Mn dopant concentration (Mn/Cu from 8 to 32), the PL emission could be tuned from white to reddish-white. Mn-doped CGZS QDs were successfully used as an emitting layer in QD-LEDs. Surprisingly, the Mn-related emission was almost quenched in the emitting layer, and only the host defect-related emission could be observed, likely due to the unbalanced carrier injection into the QD emitting layer.

The Mn-doping of CGZSe was also investigated for white-light emission. Mn-doped CGZSe QDs were prepared using the hot-injection method followed by ZnSe shelling [86]. Undoped CGZSe/ZnSe QDs show a strong PL emission at 555 nm (PL QY of 72.6%), related to the Cu deficiency. Depending on the Cu/Ga/Zn molar ratio used for the preparation of Mn-doped QDs, either a double emission (Cu and Mn-related) or a single emission at 630 nm could be observed. In the latter case, Mn^2+^ ions do not act as luminescent centers, and the emission only originates from Cu defects, as demonstrated by the PL lifetime measurements. The position of Cu-defect states relative to the Mn ^6^A_1_ state governs the PL emission of these nanocrystals. Mn-doped CGZSe QDs overcoated with several ZnSe shells and exhibiting three emissions (Mn d-d state at 590 nm, Cu-related at 500 and intrinsic emission at ca. 430 nm) were successfully used for white-light-emitting LEDs.

By using the hot-injection method, DDT as a solvent and S source and by varying the Cu/Ga from 1/4 to 4/1, CGZS QDs emitting from 520 to 619 nm could be prepared [87]. CGZS QDs exhibit a diameter of ca. 5 nm and PL QYs up to 48%. The yellow-emitting CGZS QDs (Cu/Ga = 1/1.5) were used for the fabrication of white QD-LEDs upon association with an InGaN-based blue-emitting LED.

More recently, the preparation of CGZS QDs was reported using a similar synthesis method using CuI, GaI_3_, Zn(OAc)_2_ and S as precursors and OAm and DDT as solvents and capping ligands [88]. By varying the Cu/Ga molar ratio, QDs exhibiting a small size (ca. 2.3–2.4 nm) and high-PL QYs (up to 73% for the Cu/Ga ratio of 1/10) could be prepared. Results obtained using CGZS QDs dispersed in an ethylene-vinyl acetate (EVA) copolymer as a luminescent downshifting layer in QDSSCs were rather disappointing due to the light scattering of agglomerated QDs. However, good results could be obtained using CGZS QDs@EVA in luminescent solar concentrators.

The shelling with ZnSe of CGS QDs was also investigated to increase the visible light absorption [89]. Moreover, the type-II band alignment between CGS and ZnSe QDs facilitates the charge separation and transfer. The average diameter of CGS/ZnSe QDs is ca. 7.8 nm and their PL emission is located at 590 nm. The CGS/ZnSe QDs with optimized shell thickness allowing for non-radiative recombination suppression and extended exciton lifetime were used as light absorbers to engineer photoanodes used in photoelectrochemical (PEC) H_2_ production cells. Under 1 sun illumination, the PEC device showed a photocurrent density as high as 3.5 mA·cm^−^^2^ and was demonstrated to be stable.

**Figure 17:**
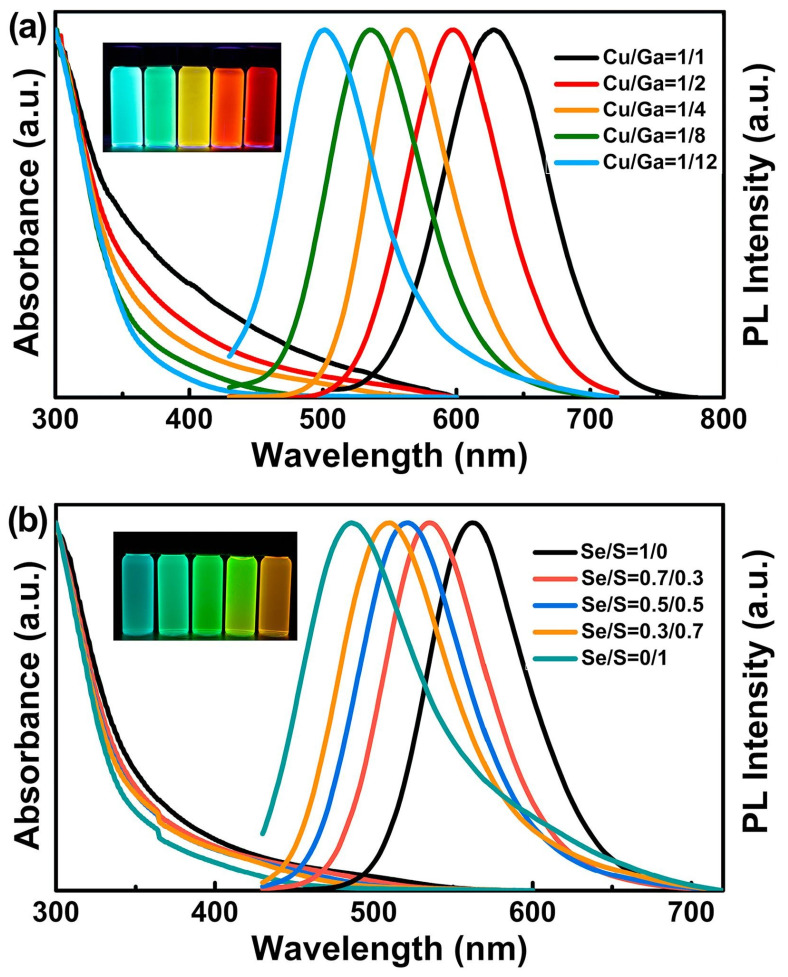
UV–vis absorption (left) and PL spectra (right, λ_ex_ = 405 nm) of (**a**) CGSe/ZnSe and (**b**) CGSe_x_S_1−x_/ZnSeS QDs prepared under various Cu/Ga and Se/S precursor molar ratios, respectively. Inset: digital photograph of samples under the radiation of a 365 nm UV lamp. Used with permission from ref. [90]. Copyright 2018 Elsevier.

The PL tunability of CGZSe QDs was first demonstrated by cationic alloying (variation of the Cu/Ga ratio) and by anionic alloying by introducing S into the CGSe core using DDT as an S source (Figure 17). With the increase in the S/Se molar ratio, the content in the wider bandgap Ga_2_S_3_ in the alloyed QDs increases, leading to a blueshift of both UV-visible absorption and the PL emission [90]. DDT forms bridged complexes with Cu and Ga, and these complexes decompose at ca. 240 °C and react with Se to generate the CGSe cores containing various amounts of S. The size of the obtained nanocrystals could be tuned from 4.2 to 5.7 nm by varying the amount of OAm during the synthesis.

The PL tunability of this family of QDs was further demonstrated by the development of CGZSe_1−x_S_x_ QDs prepared using the hot-injection method using Se-diphenylphosphine (Se-DPP) and S-DDP as highly reactive anionic sources [91]. By varying either the Cu/Ga molar ratio or the S/Se ratio, CGZSe_1−x_S_x_ QDs emitting from the blue to the red region and with PL QYs up to 73% after ZnS shelling could be prepared. Blue CGZS/ZnS (472 nm), green CGZSe_0.__3_S_0.__7_ QDs (540 nm) and red CGZSe QDs (629 nm) were selected for the fabrication of white QD-LEDs. The color-rendering indexes (CRIs) could reach 87–90, which is a remarkable value for white non-Cd QD-LEDs.

### 4.13. Cu-Ga-Al-S QDs

Cu-Ga-Al-S (CGAS) QDs have recently emerged, mainly to reduce the production costs of CuGaS_2_ QDs by substituting the expensive Ga with low-cost Al. CGAS QDs were prepared using a hot injection of S into a solution of CuI, GaI_3_ and AlCl_3_ in OAm and DDT [92]. As previously described, the Cu content allows us to tune both the UV-visible absorption and the PL emission spectra from 478 to 578 nm. The dots obtained after ZnS shelling exhibit high-PL Qys (up to 91%) and a large Stokes shift (−0.81 eV). After integration into a copolymer matrix composed of lauryl methacrylate and ethylene glycol dimethylacrylate, CGAS QDs were used to engineer luminescent solar concentrators (10 × 10 × 0.15 cm^3^) delivering a high power-conversion efficiency (PCE) of 4.29% under 1 sun AM1.5G illumination.

### 4.14. CuAlS_2_ QDs

CuAlS_2_ QDs can be prepared by the thermal decomposition of CuCl, Al(acac)_3_ and S in ODE using OA and DDT as ligands followed by their shelling with CdS [93]. CuAlS_2_ is a wide-bandgap semiconductor (*E_g_* = 3.49 eV) but forms a type-II heterojunction with CdS in which electrons are localized in the CdS shell. This allows the PL emission to be located in the visible and the NIR region (from ca. 540 to 775 nm). The core/shell CuAlS_2_/CdS show Stokes shifts higher than 100 meV originating from the marked type-II offset between CuAlS_2_ and ZnS, allowing for optical transparency, high-PL QY (up to 63%) and long PL lifetimes (ca. 1500 ns). These transparent emitters were demonstrated to be of high potential for LEDs.

The same authors investigated the electron dynamics in core/shell CuAlS_2_/ZnS QDs and demonstrated an ultrafast electron transfer (560 fs for 0.4 exciton per dot) to the surface of the dots that effectively competes with the non-radiative multi-exciton decay [94]. The photocatalytic properties of CuAlS_2_/ZnS QDs were demonstrated by the reduction of HCO_3_^−^ into formate, acetate and methanol.

More recently, the thermal decomposition method used to prepare CuAlS_2_ QDs was optimized through experiment design and theoretical investigation. Results demonstrate that a high PL intensity could be achieved by controlling the AlCl_3_ precursor concentration, confirming that a donor-acceptor pair formed by a Cu vacancy, and Cu substituted by Al is responsible for the PL emission [95]. The highly photoluminescent CuAlS_2_ QDs were used for bio-imaging.

### 4.15. CuFeS_2_ QDs

Due to the small bandgap of 0.5–0.6 eV of CuFeS_2_ in the bulk state, CuFeS_2_ QDs have gained wide attention for applications like sensing, bio-imaging and photocatalysis. Using the hot-injection method, high-quality CuFeS_2_ QDs with tunable bandgaps from the red to the NIR region could be prepared from Cu(oAc)_2_, FeCl_2_ and S using DDT and oleic acid (OA) as capping ligands [96]. A high PL could only be detected after shelling with CdS at a low temperature to avoid the alloying between CuFeS_2_ and CdS (PL QY up to 87%).

The hot injection of S into a mixture of Cu(OAc)_2_ and FeCl_2_ in a DDT/OAm mixture was also used for the preparation of CuFeS_2_ QDs. ZnS was used for the shelling due to the weak lattice mismatch (2.1%) with the CuFeS_2_ core [97]. The NIR-emitting (840 nm) QDs obtained exhibit a PL QY of 52%, an average PL lifetime of ca. 7 µs and a size of 8.9 nm, which makes them of high potential for bio-imaging. After the aqueous dispersion of CuFeS_2_/ZnS QDs by loading them into a phospholipid layer of liposome and further decorating with an isolated macrophage membrane, the nanohybrids were successfully used for in-vivo NIR fluorescence imaging.

Similar synthetic protocols were used for the preparation of CuFeS_2_ QDs used as fluorescent probes for the selective detection of Cu^2+^, Fe^3+^ and Cr_2_O_7_^2−^ ions in aqueous solution [98,99]. CuFeS_2_ QDs dispersed in water using poly(styrene)-co-maleic anhydride were also associated with poly(ethylene imine)-capped Au nanoparticles to develop an electrochemiluminescence immunosensor for the detection of cyclin D1, a protein overexpressed in numerous cancers [100].

A one-pot thermal-decomposition method using Fe(OAc)_2_ and Cu(OAc)_2_ as starting materials was also reported for the synthesis of CuFeS_2_ QDs anchored in a carbon frame. The CuFeS_2_@C composites were tested as anode materials for Li-ion batteries. A high reversible capacity (760 mA h g^−1^) for as long as 700 cycles was observed [101].

Due to the “mixed redox couple” of Cu(I)-S-Fe(III) cations contained in their crystal structure, CuFeS_2_ QDs were found to be excellent electron donors and were used as photocatalysts under visible or simulated solar light irradiation for the reduction of Cr(VI) into Cr(III) [102]. Mixed-valence single-atom Ag(I) and Ag(0) were also combined with CuFeS_2_ QDs, allowing for a bandgap decrease to 1.21 eV. Due to the electron capture properties of Ag, the charge-carrier separation was markedly improved. The Ag/CuFeS_2_ photocatalyst exhibits high activity for the synergistic reduction of Cr(VI) into Cr(III) and the degradation of the Rhodamine B dye [103]. Recently, the same group reported the decoration of CuFeS_2_ QDs with Pt for enhanced charge-carrier separation and thus improved photocatalytic activity [104].

Finally, it should be noted that the partial substitution of Fe by Al was demonstrated to be of high potential for thin-film-based solar cells. CuAl_0.25_Fe_0.75_S_2_ films show an efficiency of 3.36% [105].

## 5. Conclusions and Future Perspectives

In this review, we focus on the recent developments of several I-III-VI_2_ QDs. Synthetic methods, optoelectronic properties and the use of these QDs as light-harvesting materials for QDSSCs are discussed. Moreover, I-III-VI_2_ QDs have recently gained attention due to their low toxicity compared to II-VI group nanocrystals; their adjustable bandgap by varying their chemical composition; their high tolerance to off-stoichiometry; their solution processability; and their high stability. Moreover, due to their large absorption coefficients and high-conduction band energy, I-III-VI_2_ QDs were demonstrated to be of high potential for QDSSCs. The highest PCE value reported to date is 15.20% for Cu-In-Zn-Se-S QDs [77].

Table 1 summarizes the main characteristics (*J_SC_*, *V_OC_*, *FF* and *PCE*) of the QDSSCs described in this article and demonstrates the high potential of I-III-VI_2_ QDs for this application.

However, much progress needs to be made before I-III-VI_2_ QDs-sensitized solar cells really compete with silicon-based solar cells, which represent approximately 95% of the modules sold today.

The performance of QDSSCs and the PCE still needs to be improved. Some of the most promising avenues explored to date for QDs include:

- Further improving the light-harvesting capacity of QDs through the development of new materials.
- Decrease the density of defect trap states in these nanocrystals by tuning their chemical composition, for example, by cation and/or anion alloying, which is a key parameter for the optimal electron transfer in QDSSCs.
- Develop doped I-III-VI_2_ QDs to enhance the lifetime of trapped electrons.

The architecture of photovoltaic cells also needs to be improved by:

- Increasing the QD loading on TiO_2_. This will allow us to decrease the thickness of the QDs-sensitized photoanode and thus improve the absorption of incident photons. A decrease in the thickness of the photoanode will lead to a short transportation path of photo-generated electrons and thus limit undesirable charge recombination. Moreover, if only a small part of the TiO_2_ film is not covered by the QDs, the probability of photogenerated electrons being trapped by the redox couple in the electrolyte will also decrease, and this will markedly improve the fill factor *FF* and thus the PCE of the QDSSCs.
- Growing QDs on mesoporous TiO_2_ films using the successive ionic layer adsorption and reaction (SILAR) process to further enhance QD loading on TiO_2_ and thus the efficiency of the electron transfer from QDs to TiO_2_ [106].
- Improving the band alignment and decreasing the surface state between QDs and TiO_2_ to decrease the recombination rate at the interface.
- Gaining better knowledge of the interface charge-transfer processes not only between the TiO_2_ film and QDs sensitizers but also at the counter electrode.
- Developing solid-state cell device architectures to avoid the deterioration associated with liquid electrolytes. In this context, the deposition of a buffer layer between mesoporous TiO_2_ and the QDs (for example, In_2_S_3_, In_2_Se_3_, or CuS) was demonstrated to be effective [107,108].

## Figures and Tables

**Table 1:** Characteristics and PV performances of I-III-VI_2_ QDs in QDSSCs.

QDs	*J_sc_* (mAcm^−2^)	*V_OC_* (mV)	*FF* (%)	*PCE* (%)	Ref.
CISeTe	17.40	400	44	3.10	[41]
CISeTe	11.70	683	51	3.75	[42]
TiO_2_@CIGS	18.44	767	53	7.51	[50]
CIGSeS	13.96	260	28	1.02	[51]
CIGSe	15.27	762	69	8.02	[52]
CIGSe	0.24	432	54	0.05	[53]
CIGSe	25.01	740	62	11.49	[54]
CIGSe@rGO	8.78	690	33	2.00	[55]
AGZS	12.31	510	62	3.81	[69]
AGZSSe	14.20	540	64	4.91	[70]
CISeS	10.50	550	80	3.45	[74]
CIZSeS	25.51	780	72	14.4	[76]
CIZSeS	26.30	802	71	15.20	[77]
CIZSeS	19.50	590	55	6.40	[78]
ACISe	12.86	520	63	4.24	[82]
CGAS/ZnS	11.49	n.p.	n.p.	4.29	[92]
CuAl_0.25_Fe_0.75_S_2_	12.57	620	47	3.65	[105]

n.p.: not provided.
